# A review of the historic and present ecological role of aquatic and shoreline wood, from forest to deep sea

**DOI:** 10.1002/brv.70117

**Published:** 2025-12-21

**Authors:** Jon Dickson, Ellen Wohl, Laura L. Govers, Oscar Franken, Tjeerd J. Bouma, Han Olff, Britas Klemens Eriksson, Maryann S. Watson, Luísa M.S. Borges, Tjisse van der Heide

**Affiliations:** ^1^ Department of Coastal Systems Royal Netherlands Institute for Sea Research (NIOZ) Landsdiep 4 Den Hoorn 1797 SZ Netherlands; ^2^ Conservation Ecology Group Groningen Institute for Evolutionary Life Sciences (GELIFES), University of Groningen Landleven 1 Groningen 9747 AA Netherlands; ^3^ Department of Geosciences Colorado State University 200 W. Lake Street Fort Collins CO 80523 USA; ^4^ Department of Estuarine and Delta Systems Royal Netherlands Institute for Sea Research (NIOZ) Korringaweg 7 Yerseke 4401 NT Netherlands; ^5^ Department of Physical Geography, Faculty of Geosciences Utrecht University Princetonlaan 8a Utrecht 3584 CB Netherlands; ^6^ L3 Scientific Solutions Runder Berg 7E Geesthacht 21502 Germany

**Keywords:** driftwood, shellfish, hard substrate, large wood, river, estuary, ecosystem engineer, ocean, marine restoration, marine ecology

## Abstract

The ecology of forests, their losses, and terrestrial wood decomposition dynamics have been intensively studied and reviewed. In the aquatic realm, reviews have concentrated on large wood (LW) in rivers and the transition from freshwater to marine environments in the Pacific Northwest of North America. However, a comprehensive global synthesis of LW dynamics, including decomposition processes and human influences across the freshwater–marine continuum, is lacking. Here, we review the role of LW and its fate across the entire freshwater‐to‐marine gradient and synthesise our findings in an integrative conceptual overview. LW has been exported by rivers to sea for hundreds of millions of years. During this journey, LW acts as an ecosystem engineer by modifying its environment and the landscape. In rivers, LW alters hydrodynamics, resulting in sediment retention and changes to riverbed and shoreline morphology. Along coastlines, driftwood initiates dunes, prevents erosion, retains moisture, and provides lignocellulose‐based nutrients. Important habitats provided by floating rafts and sunken ‘islands’ of wood are found across estuarine, shelf and open/deep seas. Wood degradation gradually transitions from mechanical to biomechanical and chemotrophic. In rivers, degradation is primarily mechanical due to abrasion and impacts. In estuaries, salinity increases, allowing marine wood borers to begin biomechanical degradation; their activity remains the main degradation cause across marine environments. On the seafloor, chemotrophic micro‐organisms finalise decomposition of small fragments. LW accumulations act as biodiversity hotspots across the freshwater‐to‐marine gradient. River communities rely on induced abiotic changes such as meanders, pools, and riffles, while log jams and dams serve as velocity and predation shelters, and create pools with cooler, deeper water. The wood itself acts as attachment substrate for eggs and larvae. From estuaries seaward, the focus fully shifts to LW itself: driftwood provides lignocellulose for wood‐boring organisms and stable substrate for sessile animals and macroalgae. In shelf seas and open oceans, floating LW rafts provide shade, shelter, and attachment substrate. Humans have greatly decreased export of LW from river to sea by clearing forests for agriculture and urbanisation, damming rivers, and removing LW ‘debris’ that is often deemed a hazard or nuisance in developed areas. Indeed, the annual export of LW >3 m long to marine environments has decreased by 5,000,000 m^3^ compared to the pre‐landscape‐domestication period. Any wood that reaches the sea washes up on shore or sinks, where it is often removed by bottom trawling. Restoring historic levels of LW is implausible, but reintroductions can restore ecosystem functions along the freshwater‐to‐marine gradient. Thus far, restoration research has focused on freshwater systems, while such work is in its infancy in coastal and marine environments. We argue that managers should consider incorporating LW reintroductions at scale, as a natural and cost‐effective restoration measure across freshwater and marine environments.

## I. INTRODUCTION

Today, forests cover 28% of Earth's land mass; a decline from 40% in pre‐industrial times (Wohl & Iskin, 2021). Deforestation continues in most countries due to land conversion for agriculture, urbanisation, mining, and logging (Farrokhi *et al*., 2024). The ecology of forests, their losses, and terrestrial wood decomposition dynamics have been intensively studied and reviewed previously (Harmon *et al*., 1986; Harmon, 2021). However, an integrative global overview of wood's ecological role, its decomposition, and human impacts on its journey from freshwater to brackish and marine environments is still missing. Existing reviews, while informative, primarily focus on the Pacific Northwest coast of North America (Maser, Tarrant & Trappe, 1988; Maser & Sedell, 1994). The lack of a global overview is surprising; large dead wood may be as important for ecological communities in the aquatic sphere as in the terrestrial realm. Moreover, large dead wood may act as an ecosystem engineer (i.e. an organism, or its dead remains, that significantly modifies its environment) in aquatic systems, comparable to dead shell remains (Gutiérrez *et al*., 2003). Large wood (LW), formerly known as large woody debris (LWD), is defined as dead and downed wood greater than 10 cm in diameter and at least 1 m long (Richmond & Fausch, 1995; Merten *et al*., 2013; Wohl *et al*., 2019). This review does not consider or discuss vegetative matter smaller than this definition – i.e. detached bark, small branches, leaves, fruits, grasses, shrubs, etc. – in the LW context.

The journey of wood from forest to sea is often long, irregular and uncertain. It is a natural process that has been heavily altered by human interventions such as logging, engineering rivers (Wohl & Iskin, 2021) and the near elimination of beaver (a northern hemisphere species) populations (Brazier *et al*., 2021). Beavers, along with landslides, storms, floods, and erosion can deposit large trees into streams and rivers (Gurnell, England & Burgess‐Gamble, 2019; Maser & Sedell, 1994; Stout *et al*., 2018; Wohl, 2017). Much of this LW may become trapped by, or become, snags (waterlogged and sunken LW that may protrude above the water's surface), be left stranded ashore by receding water levels, or be removed by humans before it reaches an estuary (Gonor, Sedell & Benner, 1988). LW that does reach estuaries is affected by a new suite of factors: tides, coastal currents, wind, waves, marine wood‐boring organisms, and human activity, along with the natural depositional processes that create deltas (Kramer & Wohl, 2015; Murphy *et al*., 2021). Wood that escapes rivers, estuaries, and coastal depositional processes can be transported out to sea by winds and currents (Murphy *et al*., 2021). Here, it can be colonised by marine hard substrate communities, becomes increasingly waterlogged and decreases in buoyancy (Thiel & Gutow, 2005a,b). Some of this wood escapes beyond the continental shelf to make it to the open ocean where it eventually sinks to the ocean floor (Turner, 1973). In every aquatic ecosystem it passes or remains in, LW plays a key role in creating food, shelter, spawning grounds and feeding habitat for biota. Throughout its journey to the sea, LW also provides important ecosystem services such as bank stability as well as erosion and flood control (Gurnell *et al*., 2005; Merten *et al*., 2013; Wohl, 2013; Wohl *et al*., 2019). Moreover, LW also forms a critical land–sea linkage through provisioning of nutrients (lignocellulose) and structure to marine environments. Given the human‐driven decrease in LW export (Wohl & Iskin, 2021), its functions and services provided to rivers, brackish, and marine systems have greatly diminished.

There is more literature relating to LW in freshwater environments than to LW in estuaries, shelf seas, and open ocean/deep sea. In rivers, LW dynamics are rather well studied (Gurnell *et al*., 2005; Maser *et al*., 1988; Maser & Sedell, 1994; Wohl *et al*., 2019). However, beyond rivers, the fate and role of LW are less understood. In estuaries, some work exists as to its role on beaches, mudflats, and tidal marshes in the Pacific Northwest of North America, while studies on sunken LW are lacking (Gonor *et al*., 1988, Maser & Sedell, 1994). Knowledge of wood in shelf seas largely focuses on coastal morphology–wood (Murphy *et al*., 2021) and infrastructure–wood interactions (Doong *et al*., 2011). Here, knowledge on the biological role of sunken wood is patchy in space and time, and mostly concerns artificial reefs (Alam *et al*., 2020; Dickson *et al*., 2023; Masuda *et al*., 2010), and shipwrecks (González‐Duarte *et al*., 2018). Open sea literature largely concerns rafting (Thiel & Gutow, 2005a,b) or transport of wood (Murphy *et al*., 2021). Studies regarding woodfalls in the deep sea typically revolve around deep sea bottom trawls (Distel *et al*., 2000; Fagervold *et al*., 2012; Turner, 1973; Wolff, 1979) or intentional wood placement experiments (Bienhold *et al*., 2013; McClain & Barry, 2014; McClain *et al*., 2023; Romano *et al*., 2020, 2014; Voight, Heck & Du Clos, 2023). Finally, most research on LW in aquatic environments focuses on the northern hemisphere, where >57% of global forests (Pan *et al*., 2013), 74% of land area (Boggs, 1945), and 88% of the human population is found (Kummu & Varis, 2011). These geographic and demographic patterns likely contribute to a temperate‐north literature bias covering LW in aquatic environments.

**Fig. 1:**
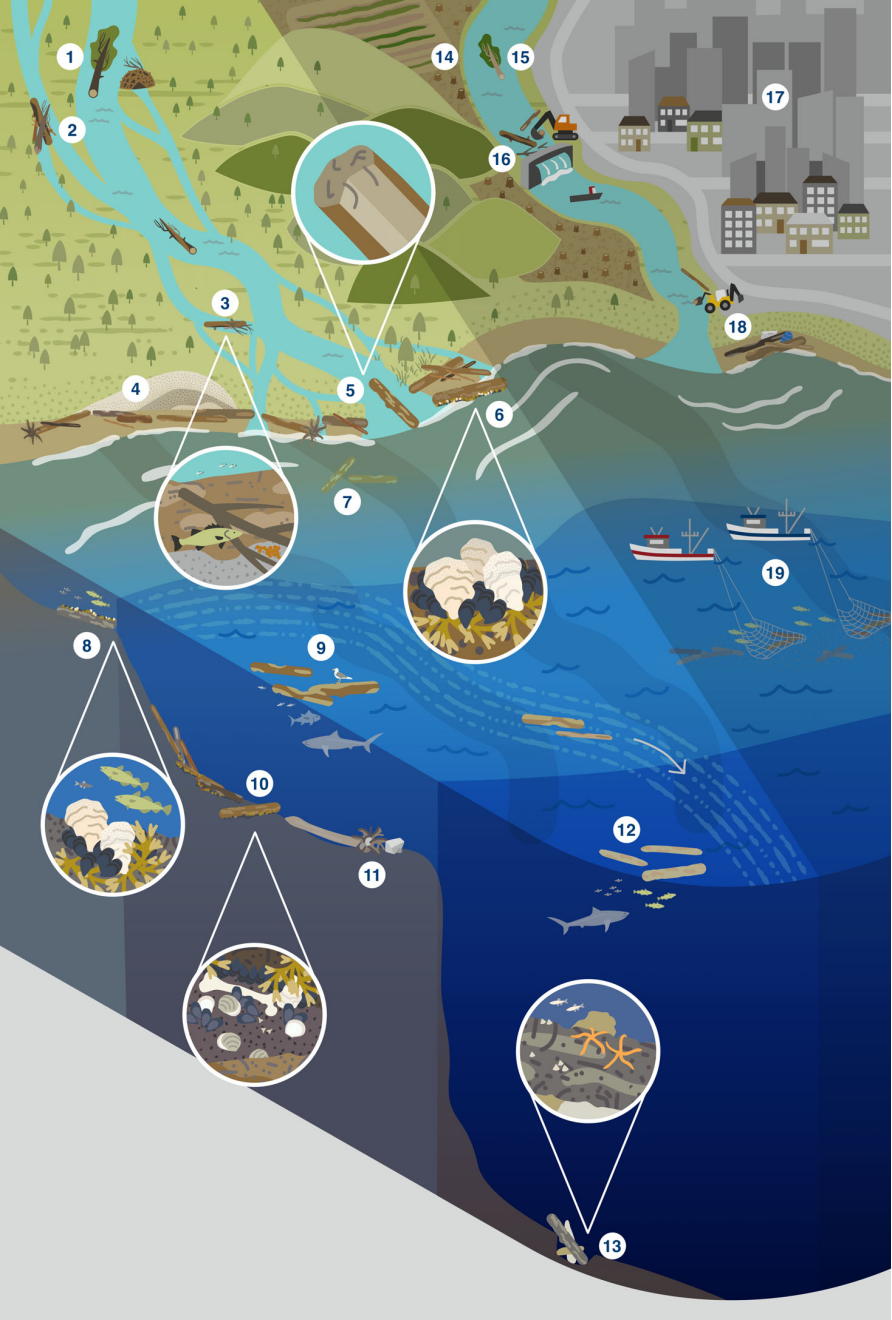
Summary of the ecosystem roles of large wood (LW) across the freshwater to marine gradient, and human impacts on these processes. 1 = tree enters watercourse. 2 = log jams create side channels and wetlands. 3 = LW creates lee in flow, gravel accumulates/fish save energy. 4 = LW accumulates sand and initiates dunes. 5 = brackish estuary allows some shipworm, pholad, and pill bug (the latter two in warm waters) colonisation. 6 = LW provides shellfish and seaweed substrate, and habitat for fish. 7 = sunken wood collects around river mouths. 8 = LW provides shellfish and seaweed substrate, and foraging grounds for fish. 9 = LW rafts provide floating habitat for e.g. birds and fish. 10 = LW creates accumulation zone of shells, bones, peat, and other wood. 11 = LW moves stones locked in root wads to sea, initiating wood/stone reefs. 12 = floating LW provides shelter and foraging grounds for fish, and attachment points for shellfish. 13 = LW tends to collect in gullies and canyons, providing nutrients and substrate. 14 = forests cleared for agriculture. 15 = LW enters watercourse from upstream. 16 = dam operation traps and removes wood. 17 = urban development has replaced forest. 18 = wood on beaches removed. 19 = bottom trawlers remove sunken wood/shellfish reefs/other substrate/fish.

Here, we review the global ecological role of LW, its decomposition, and removal across the entire fresh‐to‐marine gradient (Fig. 1). We distinguish four distinct systems along this gradient: streams and rivers, estuaries, shelf seas/beaches and open/deep seas. Lakes are omitted, but for an overview of lake LW, see Czarnecka (2016). Within each of these four systems, we review the ecological role of wood, its degradation, and highlight how human actions have affected natural processes involving LW. Finally, we synthesise our findings in a global integrative conceptual overview of the ecological roles of wood across the freshwater‐to‐marine gradient.

## II. STREAMS AND RIVERS

From mountain streams to lowland rivers, natural watercourses shape the landscape (Williams & Morris, 2005). As rivers meander and undercut their banks, trees fall into the water, which then moves the woody material downstream. On developed rivers with fixed, hardened banks, this natural undercutting rarely occurs. However, both developed and natural rivers can receive LW through multiple processes. These include bank erosion; upland debris flows, landslides, or avalanches; individual tree mortality or mass mortality from wildfire, insects, or blowdown; and exhumation of wood previously buried in the floodplain. The spatio‐temporal differences in methods and volumes of wood recruitment, transport, and storage along rivers give rise to the concepts of wood budgets (Benda & Sias, 2003; Benda *et al*., 2003), wood process domains (Lawrence, Resh & Cover, 2013), and wood regimes (Wohl *et al*., 2019) that describe longitudinal differences in wood dynamics across a river network and along the length of a river. LW in rivers has been well synthesised before in multiple reviews and books (e.g. Collins *et al*., 2012; Gurnell *et al*., 2019; Maser & Sedell, 1994; Wohl *et al*., 2019); this section merely provides context and framing for the first stage of the journey of LW from rivers to sea.

### (1) Rivers – transport, fate and degradation

Wood enters watercourses year‐round, although input rates are generally higher during winter, spring freshet, or high‐energy events such as atmospheric rivers, hurricanes, or tropical rainy seasons (Doong *et al*., 2011; Phillips & Park, 2009; West *et al*., 2011). In seasons or events with high precipitation, increased water levels cause more bank erosion from faster, fuller rivers (Oswald & Wohl, 2008). Additionally, landslides and other mass wasting events such as debris flows, rock avalanches, solifluction, and bank slumping are more common as soils saturate; these events introduce more LW into rivers already running with increased energy and water levels (Ruiz‐Villanueva *et al*., 2014, Bauer *et al*., 2013).

The inherent mobility of wood, or lack thereof, is nuanced and system dependent. Single pieces or masses of wood can move downstream suspended or in contact with the channel bed (Braudrick *et al*., 1997; Ruiz‐Villanueva *et al*., 2019). Field experiments and flume studies indicate that LW mobility is governed by both flow characteristics and piece characteristics. Relevant flow characteristics include flow width, depth, duration, rate of rise and fall, and history of wood‐transporting flows relative to wood supply (Kramer & Wohl, 2017). Most wood is transported during relatively infrequent high flows, but flows under bankfull can transport up to 30% of stored wood (Kramer & Wohl, 2017). Relevant piece characteristics include wood density, piece size relative to channel width and flow depth, piece complexity (i.e. presence of root wads and branches), piece orientation relative to flow direction, and the trapping characteristics (Merten *et al*., 2010; Ruiz‐Villanueva *et al*., 2016a,b; Chen *et al*., 2020). The most mobile wood comes from more buoyant, small pieces that are simple cylinders resting against the channel boundaries rather than pinned against other obstacles, incorporated in a jam, or partially buried in the bed, or partly resting above the bankfull channel. Buoyant wood remains mobilised until trapped against obstacles, stranded at the high water line, or washed ashore (Gonor *et al*., 1988; Gurnell *et al*., 2019; Wohl *et al*., 2019). Often, this wood will be re‐entrained by subsequent high‐water events so that it undergoes repeated periods of stability and decay interspersed with periods of transport, abrasion, and breakage, which Kramer & Wohl (2017) likened to nutrient spiralling.

Abrasion from impacts is the main degradation mechanism of riverine wood; these impacts occur against other aquatic wood, scouring by sand and gravels, and impact with riverbanks, bottoms, and infrastructure such as bridge pylons as LW travels through river systems (Gurnell *et al*., 2019; Merten *et al*., 2013; Murphy *et al*., 2021). While sturdier branches and root wads (the large, sturdy ‘ball’ of roots at the base of a tree) can remain when LW reaches the mouth of the river, mechanical stress and impacts generally cause wood to lose most of its bark and branches, provided that the journey is long enough (Murphy *et al*., 2021; Steelandt *et al*., 2015). For example, driftwood found on Arctic coastlines tends to have less complexity due to loss of root wads and branches during transit through long Arctic rivers with abundant abrasive ice (e.g. the rivers Mackenzie and Ob) and interactions with sea ice when contrasted with temperate‐sea LW (Murphy *et al*., 2021), where rivers are generally shorter and river‐exported LW does not usually interact with river or sea ice. Driftwood that has been washed ashore while still retaining complex elements of root wads and branches is presumably near its source river and has not undergone significant battering and abrasion against coastlines by waves (Murphy *et al*., 2021). Regardless of climatic region, the primary mechanism behind degradation of LW in river channels is mechanical (breakage/scouring) and concurrent and/or subsequent mobilisation (Merten *et al*., 2013, Anderson, 1989). Fine twigs and branches break off at a proportionally greater rate than LW abrades (Murphy *et al*., 2021). LW generally has the strength to withstand more severe impacts, for example, against other wood, stones, or bridges as evidenced by large unbroken driftwood piled in significant masses against bridge pylons (Ockelford *et al*., 2024; Schmocker & Weitbrecht, 2013). Decay of LW may be equally important as mechanical damage in floodplain environments when considering LW degradation.

Next to abrasion, biotic decomposition is a secondary wood degradation process and occurs *via* microbial activity, particularly fungi, and to a lesser extent from feeding by invertebrates and fishes (Harmon *et al*., 1986). Once wood is fully waterlogged, however, it decays very slowly as fungi are virtually absent (Maser *et al*., 1988); degradation occurs by invertebrate borers, scrapers, and microbial activity (Dolloff & Melvin, 2003). In addition, wood‐grazing fish can convert wood and associated microbes into energy in some tropical rivers (Lujan, German & Winemiller, 2011). Yet, the overall role of wood‐consuming (micro‐)organisms is limited compared to terrestrial and marine environments (Anderson, 1989; Benke & Wallace, 2003). Wood that first rots in a terrestrial environment is consumed by burrowing and scraping organisms much more quickly than intact, ‘barked’ wood once entering the river (Anderson, Steedman & Dudley, 1984). White‐rot fungi degrade lignin and cellulose, whereas soft‐rot and brown‐rot fungi degrade only cellulose (Harmon *et al*., 1986). White and brown rot drives rapid decay in damp but not saturated wood, while soft rot slowly degrades wet and saturated wood (Goodell, Qian & Jellison, 2008). Hence, rot can be important on floodplains with repeated submergence and exposure. However, once wood is fully submerged, rot is generally not a significant degradation factor over short to medium timescales (Wohl, 2013).

Long‐term decay plays a part in freshwater wood degradation, although it is highly dependent on the tree species and the climate due to interacting factors of temperature, moisture and air exposure. Complete decay of riverine wood ranges from potentially hundreds of years in drier temperate regions to less than a decade in tropical riverine systems (Harmon *et al*., 1986; Merten *et al*., 2013). Due to the higher surface area to volume ratio of smaller pieces, decay affects small wood at a greater rate than LW. A study in Minnesota, USA by Merten *et al*. (2013) found that small complex wood decayed 9.4% by mass within 120 days whereas LW was reduced by 1.9% over one year. Permanently submerged LW may remain solid and stable from centuries to millennia (Wohl, 2013): this statement is given as a generality, as geography, water temperature, climate, flow speed, wood species, softwood *versus* hardwood, and state of decay of LW will all affect this. Examples of relatively undecayed buried or submerged wood in substantial quantities come from eucalypt species in Australia (O'Connor, 1992), and bald cypress (*Taxodium distichum*) and longleaf pine (*Pinus palustris*) logs in the southeastern USA (Kaeser & Litts, 2008). The relatively undecayed state of this wood primarily reflects decay‐resistant tree species. Finally, photodegradation *via* sunlight can play a role in freshwater wood degradation, albeit minor. For example, photodegradation was found to degrade about 6 mm off the surface layer of a log over 100 years (Murphy *et al*., 2021). This can create greater rugosity at the wood surface, allowing increased abrasion and/or chemotrophic degradation of LW through an increase in surface area (Murphy *et al*., 2021).

In riverine environments, softwoods are preferred over hardwoods for wood replacement and restoration projects as they exhibit greater long‐term resistance to decay (Bilby *et al*., 1999; Dolloff & Melvin Jr., 2003). However, Bilby *et al*. (1999) note that both last for decades when fully submerged. Indeed, the faster decay rate of hardwoods occurs when exposed to oxygen during emergence and is linked to their greater nutritional profile in the sapwood and bark (Bilby *et al*., 1999; Harmon *et al*., 1986). Furthermore, softwoods are generally larger in size and thus preferred for in‐stream structures (Bilby, 1984). Interestingly, this is reversed within marine environments, where hardwoods are preferred for building and infrastructure (Borges *et al*., 2008; Borges, Cragg & Williams, 2003; Williams *et al*., 2018).

### (2) Rivers – ecological role

Riverine wood creates stable attachment surfaces for biofilms and invertebrates and calm water zones where fish, amphibians and insects lay eggs on the wood (Anderson *et al*., 1984; Benke & Wallace, 2003). Snags in rivers serve as invertebrate biodiversity hotspots (Benke & Wallace, 2003; Magliozzi *et al*., 2020), and many snag‐dependent animals remain partially or fully within the riverine food web throughout their lifecycle. However, the main effects of riverine wood are changes to the physical river profile, such as increased pool volume (Richmond & Fausch, 1995; Martens & Devine, 2023), increased hyporheic exchange flows (Sawyer & Cardenas, 2012; Gambill *et al*., 2025), and secondary channel formation (Collins *et al*., 2012; Marshall & Wohl, 2023), in turn influencing biotic communities (Benke & Wallace, 2003; Dolloff & Melvin, 2003; Stoffers *et al*., 2022; Wohl, 2013; Wohl *et al*., 2019).

Stuck single logs and log jams alter the physical and biological characteristics of the stream (Collins *et al*., 2012; Marshall *et al*., 2024). Log jams can create lees, which act as high‐flow‐velocity shelters, as well as deep, cool pools (Maser *et al*., 1988); both are preferred habitat over riffles and channels for many fish (Lester & Boulton, 2008), particularly during spring freshet (Reinhold *et al*., 2016). Indeed, logjams in rivers can account for 65% of flow resistance in forested basins, increasing to 75–98% where LW has induced a change in water profile elevation (e.g. rapids) (Dixon, 2013; Bao *et al*., 2024). Both beaver dams and natural log jams, therefore, slow river flow. Lees created by log jams and beaver dams promote sediment and gravel deposition, which is a key environmental consideration for fish that require gravel/sand for egg laying, i.e. salmonids. Similarly, sunken logs create subsurface low‐flow ‘wakes’ where fish often reside to conserve energy (Hafs *et al*., 2014). LW accumulations also provide cover for fish and other aquatic animals (D'Aoust & Millar, 2000) as well as keeping the river cooler by providing shade (Lester & Boulton, 2008).

While individual or small numbers of intertwined LW create in‐stream habitat, log jams can alter the course of a stream (Bisson, Sullivan & Nielsen, 1988; Collins *et al*., 2012; Marshall *et al*., 2024; Maser *et al*., 1988). Specifically, large log jams can create dams that divert flow, split or redirect a channel, focus fluvial erosion, or enhance overbank flow (Wohl, 2013). They also create larger backwater and scour pools and store large amounts of particulate organic matter (Livers & Wohl, 2021; Welling, Wilcox & Dixon, 2021). LW jams often create or maintain side channels (Bisson *et al*., 1988; Maser *et al*., 1988) with lower energy conditions than the main channel, yielding important spawning and foraging grounds for fish. LW obstructions also increase the diversity of hydraulic conditions, water temperature and chemistry for microbial communities and invertebrates (e.g. Stoffers *et al*., 2022). Furthermore, large woody obstructions induce turbulence, increasing water (Lester & Boulton, 2008) and upper sediment oxygen levels (Wilhelmsen *et al*., 2021). Finally, by obstructing and diverting water into side channels and floodplains, LW slows streams and lessens erosion (Lester & Boulton, 2008). Indeed, it was found that removing well‐anchored LW can reduce the stability of the channel bed and banks, as well as habitat quality (Toews & Moore, 1982; Brooks, Brierley & Millar, 2003).

Beavers have been creating their own log dams by felling trees into rivers and lakes in the Northern Hemisphere for 20 million years (Plint *et al*., 2020). Indeed, a single beaver can add several tonnes of wood to streams annually, magnitudes more than the addition from deadfall (Maser & Sedell, 1994). The North American (*Castor canadensis*) and Eurasian (*Castor fiber*) beaver inhabit broad swaths across northern boreal and temperate forests, where they rely on riparian woody vegetation for food and to create shelter. As ecosystem engineers (Jones, Lawton & Shachak, 1994), beavers create or alter many natural riverine processes, such as log dams and jams, channel redirection, wetlands, and permanent water retention in areas of ephemeral rivers (Larsen, Larsen & Lane, 2021). These landscape modifications also create wetland ‘firebreaks’ that are difficult for wildfires to cross (Fairfax & Whittle, 2020). This allows vegetation to provide continued structural stability to soil on slopes and in riparian areas (Easson, 2002), thereby lessening wide‐scale sediment runoff into rivers (Dunn, Rathburn & Wohl, 2024) that can deplete oxygen levels and smother fish and their eggs (Kemp *et al*., 2011). Furthermore, beaver dams on rivers create ‘step–stair’ stream profiles that promote significant sediment retention in beaver ponds and slow flow velocity (Dixon, 2013; Naiman, Johnston & Kelley, 1988; Wohl & Scott, 2017). Indeed, small 4–18 m^3^ wood dams were found to retain 2000–6500 m^3^ of sediment (McDowell & Naiman, 1986). Moreover, these ponds host 2–5 times the biomass of unaltered riffle sites, although community species assemblage remains similar to running river sites (Naiman *et al*., 1988).

### (3) Rivers – human impact

LW in rivers has traditionally been viewed as a negative feature and has been removed for ‘*at least a century in high‐income countries and for several centuries in some regions of Eurasia*’ (Wohl, 2015, p. 168). For example, in the Murray River basin in the Australian states of South Australia and Victoria, river ‘desnagging’ (the removal of snags) commenced in 1855. It was estimated that a single snag boat removed three million snags from the Murray and its tributaries from the 1910s to 1960s (Lloyd, Walker & Hillman, 1991). Similarly, more than 1.5 million snags were removed from 30 US rivers between 1867 and 1912 (Harmon *et al*., 1986; Wohl, 2014). Simultaneous river clearing activities such as cutting overhanging trees and blasting boulders with explosives also occurred during these operations (Gonor *et al*., 1988). These examples are hardly unique but illustrate the scale of active wood removal that has been conducted for multiple centuries globally.

Alongside active removal of LW from rivers, logging has greatly reduced the amount of riverine wood. Wilderness streams running through drainage basins with natural forests have been found to hold more than 100 times as much wood as rivers running through logged basins in similar regions, even without active riverine wood removal (Wohl *et al*., 2017). Rivers running through drainage basins that had been logged were found to hold wood that was half the length (4.5 m) as unlogged basins (10 m) (Sedell *et al*., 1988). Similarly, when considering old‐growth (600 years) *versus* second‐growth (21–44 years) forested basins, rivers running through old growth held more than three times the volume of logs compared to channels in second‐growth basins; these logs were also larger (Gregory *et al*., 2024). Logging activities also indirectly affect stream dynamics; in North America, for instance, access to logging sites by road was often difficult so ‘splash dams’ were built (Wohl *et al*., 2019). These dams retained large amounts of logged timber that were released during periodic artificial freshets, sending large amounts of wood downriver to mill sites. Although this practice was largely discontinued by the 1950s, river geomorphology remains affected today (Ruffing, Daniels & Dwire, 2015): artificial freshets and mass downstream movement of timber scoured river bottoms, damaged riverbanks, straightened and widened channels, and removed gravel (Sedell, Leone & Duval, 1991). Logs bashing into each other caused the rapid removal of tonnes of bark which sank and smothered large areas of riverbed (Sedell *et al*., 1991). If logs jammed together before reaching the mill site, dynamite was used to clear them (Sedell *et al*., 1991). This practice along with the physical degradation and smothering of the riverbed proved detrimental to fish populations (Sedell *et al*., 1991).

Today, dams still collect large amounts of wood in forested watersheds (Fremier, Seo & Nakamura, 2010; Qatarneh *et al*., 2021; Ruiz‐Villanueva *et al*., 2016b; Shumilova *et al*., 2019). Generally, this is mechanically removed by heavy machinery and set aside for landfill or combustion (Qatarneh *et al*., 2021). The amount of wood trapped by dams is directly correlated to land use and drainage basin size (Shumilova *et al*., 2019). If dams do not experience water flow over the top of the dam, the role of wood in rivers and downstream environments is abruptly terminated, leading to straighter, faster rivers, thereby increasing bank erosion.

Beaver populations have been greatly reduced throughout most of their range due to over‐trapping and hunting for their fur (Naiman *et al*., 1988). Eurasian beavers, which historically ranged from Portugal to China, and south to Persia (Chu & Jiang, 2009; Halley, Rosell & Saveljev, 2012; Halley, Saveljev & Rosell, 2021; Larsen *et al*., 2021), were reduced to eight fragmented populations totalling 1200 individuals at the beginning of the 20th century (Halley *et al*., 2012); estimations of pre‐exploitation Eurasian beaver populations do not exist. While populations have recovered – in part due to active reintroduction – to an estimated 1.5 million individuals from Europe to China (Halley *et al*., 2021), this number is likely still low by historic standards. North American beavers ranged from Arctic tundra to northern Mexico (Wohl, 2021) and were estimated to number 60 to 400 million pre fur trade, but went nearly extinct in the contiguous USA by 1900 (Naiman *et al*., 1988). Estimates from the 1980s suggest current populations of approximately six to 12 million beavers (Naiman *et al*., 1988).

The role of beavers in facilitating wood entry into freshwater and estuaries cannot be overstated. With fewer beavers, rivers, streams, estuaries and wetlands recruit and retain less wood than they did historically. The loss of beavers and their dams likely means that river valleys are drier. Indeed, Maser & Sedell (1994) quote historical records (from the Pacific Northwest) stating that travel was only possible at the edges of river valleys because the valley bottoms themselves consisted of impassable beaver‐facilitated wetlands. Thus, the loss of beavers not only reduced LW recruitment but likely also changed local ecotopes – and inherent vegetation therein. Given beavers' historic range and abundance, their decline has affected many natural processes and systems along temperate Northern Hemisphere rivers. These include river and wetland‐fringing aquatic and terrestrial ecosystems, river hydrology and geomorphology, sediment retention, nutrient export, and groundwater levels; see Larsen *et al*. (2021) for further reading. For the purposes of this review, it should be understood that humans have altered the wood cycle not only by direct actions such as logging and development, but also indirectly (in the temperate Northern Hemisphere) by decreasing beaver populations.

Interest in restoring the ecological functions of wood in rivers has increased (Ockelford *et al*., 2024). Indeed, re‐introduction of LW is now successfully employed as both a habitat restoration tool and flood management intervention (Deane *et al*., 2021; Louhi *et al*., 2016). For example, placed LW can increase the population abundance, size and biomass of salmonid fish (Dolloff & Melvin Jr., 2003; Foote, Biron & Grant, 2020; Pess *et al*., 2023; Roni *et al*., 2015; Whiteway *et al*., 2010). However, due to the inconvenience or potential danger to infrastructure and shipping, restoration projects in navigable rivers must safely secure their LW (Cornett *et al*., 2017) or move to smaller side channels (Ockelford *et al*., 2024). Stakeholders of downstream bridges and dams are particularly concerned about mobilised LW used for restoration. However, secured wood does not fully mimic the natural role of mobile LW. Free‐to‐mobilise LW improves channel–floodplain connectivity (Laterell & Naiman, 2007; Wohl, 2013), provides refugia during floodplain inundation, dissipates flow energy (Juez *et al*., 2019), diversifies wood decay states (Ballinger, MacNally & Lake, 2010), and supplies LW to downstream environments (West *et al*., 2011; Wohl *et al*., 2024). Naturally, the potential hazard of loose LW leads to restrictions on the scale of reintroducing LW; replaced wood is orders of magnitude less than natural wood jams.

For example, the ‘Great Raft’ wood jam on the Red River of Louisiana, USA stretched more than 260 km along the river and persisted from the 12th century to the 1830s when it was broken up by humans (Watson, 1967). It is theorised that the wood jams that formed and naturally broke on the Mississippi and its tributaries reached the Gulf of Mexico, where they sank and provided hard substrate for vast historic oyster reefs. Firsthand accounts from an oyster aquaculture professional on logging debris tell that LW reaching the sea led to the expansion of Apalachicola, Florida, USA oyster reefs in the 1890s –1940s (Kung Li, personal communication, 2024); this is an observed modern‐day mimic of historical processes that likely occurred throughout forested watersheds globally. Re‐introducing mobile‐capable LW at natural scales is not realistic. It would take a river approximately 255 years to return passively to its natural wood load after human ‘cleaning’ (Stout *et al*., 2018) – presuming land use within the river basin exists in a natural state. Yet, some rivers still episodically export enormous amounts of wood to the ocean. This includes boreal rivers such as the Mackenzie River of Canada (Sendrowski *et al*., 2023) and steep, tropical rivers that experience episodic typhoon or earthquake‐driven landsliding (West *et al*., 2011). These rivers provide some insight into the magnitudes of wood that were historically exported from a much greater number and diversity of rivers.

## III. ESTUARIES AND NEARSHORE

Estuaries and deltas are partially enclosed coastal water bodies where one or more rivers or streams meet the sea. These transitional waters range from predominantly fresh water upstream to predominantly sea water near the sea (Bauer *et al*., 2013). In these waters, not only does the salinity change, but the currents, flow direction, and water depth also vary with tidal influence. This confluence of fresh and salt water results in some of the most productive habitats on Earth (Cardoso, 2021) that tend to be high in biodiversity. In most systems, estuaries occur synonymously with deltas (Emery & Stevenson, 1957) that are created as the widening and slowing rivers deposit large amounts of typically muddy sediments (Meade, 1972), with plumes that can extend far out to sea (Emery & Stevenson, 1957; Meade, 1972). Along with sediment, rivers also deposit larger substrates within estuaries which have been travelling as bed load within rivers, such as gravels, cobbles (Meade, 1972) and wood (Diefenderfer & Montgomery, 2009; Hinwood & McLean, 2017; Hood, 2023).

### (1) Estuaries – transport, fate and degradation

Global knowledge regarding transport and fate of natural LW in estuaries is lacking. Prior to a 2017 study in Australia (Hinwood & McLean, 2017), no quantitative studies existed, with other literature being largely descriptive (Gonor *et al*., 1988; Maser *et al*., 1988). Wind, tide, and river flow can mobilise and/or strand LW on intertidal bars and shoals; due to wind and waves, deposited wood is generally parallel with the shore (Hinwood & McLean, 2017). Since estuarine channels are typically wider and deeper, log jams are less likely than in rivers (Hinwood & McLean, 2017). That said, it has been found that maximum wood length scales positively with channel size: the wider the channel, the larger the wood (Hood, 2023). LW in estuaries contributes locally to scour and sediment accumulation, but in contrast to rivers, does not currently impact estuarine gradients or geomorphology (Hinwood & McLean, 2017). This may have been different historically when vast amounts of LW were exported to estuaries and coast. Subtidally, little is known about sunken LW other than that it tends to collect around river mouths (Schwabe *et al*., 2015).

In contrast to rivers, where wood degradation is almost exclusively mechanical in nature, LW in estuaries is also degraded by biota. In rivers, most organisms cannot directly use the nutrients in wood. This is because chemical compounds in woody substrates, such as suberin in the bark, cutins in the leaves, and especially the lignocellulose complex (an organic matrix of the polymers cellulose, hemicellulose, pectin, and lignin), are highly resistant to enzymatic degradation. Lignocellulose, an energy‐rich substrate, is particularly recalcitrant and thus prolongs the lifespan of LW (Cragg *et al*., 2015, 2020). However, a group of organisms, collectively known as marine wood borers, do feed on wood directly (xylotrophy; Distel *et al*., 2011). This group includes bivalves and crustaceans belonging to several families. The bivalves belong to the families Teredinidae (commonly known as shipworms), Pholadidae (pholads), and Xylophagaidae (xylophagaids), while the crustaceans belong to the families Limnoriidae (gribbles), Sphaeromatidae (pill bugs), and Cheluridae (chelurids). Shipworms and xylophagaids digest lignocellulose (Cragg *et al*., 2015) by hosting endosymbiotic bacteria in their gills and digestive system (Waterbury, Calloway & Turner, 1983; Distel & Roberts, 1997; Distel, Beaudoin & Morrill, 2002). These bacteria – e.g. *Teredinobacter turnerae* – produce lignocellulose‐degrading enzymes that not only effectively overcome this recalcitrance but also fix nitrogen (Lechene *et al*., 2007). In addition, shipworms and gribbles also produce endogenous enzymes that aid the digestion of lignocellulose (King *et al*., 2010; Kern *et al*., 2013; Honein *et al*., 2012). For further information on these processes, see Sabbadin *et al*. (2018), and the CAZy database (Carbohydrate‐Active enZYmes; Drula *et al*., 2022). These processes facilitate the direct transfer of terrestrial woody nutrients to wood borer biomass. Some shipworms, for instance, have astonishing growth rates, and some species may reach 30 cm (250 g) in 6 months (Willer & Aldridge, 2020; Poon, Shipway & Willer, 2025), making this transfer very effective.

In estuaries, the activity of marine wood borers is strongly constrained by lower salinity; for instance, the teredinid *Lyrodus pedicellatus* (Borges *et al*., 2014a) and limnoriids (Limnoriidae) cannot survive under such conditions (Borges, Merckelbach & Cragg, 2014b). Nevertheless, some species are able to tolerate, and even thrive in, brackish waters. In temperate waters, *Teredo navalis* (thought to be distributed near‐worldwide) is the main species feeding on wood in estuaries (e.g. Paalvast & van der Velde, 2011). In tropical waters, some teredinid species, such as *Teredo poculifer*, inhabit estuaries (Turner, 1966; Rayner, 1983). Similarly, pholads, e.g. *Martesia striata*, and pill bugs, e.g. *Sphaeroma terebrans*, also bore into wood in estuaries, but for shelter only (Eaton & Hale, 1993; Si, Bellwood & Alexander, 2002). The deterioration caused by marine wood borers also depends on wood species, with some, e.g. *Pinus sylvestris* (pine) consumed quicker than others, e.g. *Chlorocardium rodiei* (greenheart) (Borges *et al*., 2008; Williams *et al*., 2018).

Shipworms also filter‐feed by extending their siphons into the water through the entrance hole created when the larvae entered the wood (Turner, 1966). This filter‐feeding behaviour may be the reason why they are absent in highly polluted waters (Nair, 1984). More generally, abiotic factors (e.g. salinity) place a spatiotemporal limit on how far upstream marine wood borers consume wood (Paalvast & van der Velde, 2011). Cooler temperatures place seasonal limits on wood borer activity in temperate waters (Borges *et al*., 2014a,b) and polar estuaries and seas (Treneman *et al*., 2018), while wood borers feed and reproduce year‐round in tropical waters (MacIntosh, De Nys & Whalan, 2012).

Compared to wood borers, marine fungi and bacteria (chemoorganotrophs) play a smaller role in the degradation of LW in estuaries (Cragg *et al*., 2020; Pop Ristova *et al*., 2017; Wolff, 1979; Björdal, 2012). Microbial degradation is relatively more important on smaller wood than in LW due to its high surface area to volume ratio (Merten *et al*., 2013).

Like most marine wood borers, microorganisms have little effect on wood with a bark covering. As in terrestrial systems, on wood where bark remains intact, the bark continues to act as a protective barrier against bacteria, fungi, and wood borers (Kohlmeyer, Bebout & Volkmann‐Kohlmeyer, 1995). Once bark has been mechanically removed from the wood, marine fungi and bacteria start to soften the wood. This allows wood borers to move more easily into the now‐softened wood, leading to facilitated bio‐mechanical degradation. The tunnels of the wood borers subsequently provide more surface area for bacteria and fungi to colonise (Lane, 1961), which may lead to a mutualistic relationship and overall faster degradation of the wood.

### (2) Estuaries – ecological role

When washing ashore, LW functions as an agent of disturbance to communities (Simenstad & Wick, 2003). In salt marshes, stranded LW promotes localised sediment accretion (Gonor *et al*., 1988, Eilers, 1975), creating raised ‘islands’ for vegetation to establish. King tides and storm surges can refloat and move this wood, leaving flooded depressions that serve as habitat for juvenile fish (Eilers, 1975). On rocky shores, wave‐thrown LW can crush and scrape away sessile organisms to create bare patches that can become further enlarged by wave action (Dayton, 1971). Recolonisation of these bare patches facilitates the formation of a mosaic of heterogenous successional stages of sessile communities (Bulleri & Benedetti‐Cecchi, 2006; Menge, 1975).

In brackish estuarine waters, some wood‐boring species, such as *Teredo navalis* in temperate waters and *Dicyathifer mannii* in tropical waters, can tolerate the low salinity and perform their essential biological role (Borges, 2014; Rayner, 1983). Teredinids are critical both as ecosystem engineers (degrading wood and increasing surface complexity) and facilitators of land–sea nutrient linkages (Cragg *et al*., 2015; McClain *et al*., 2025). A decrease in LW reaching estuaries not only deprives these environments and associated animals of hard substrate as settlement, shelter, and habitat, but also equates to a decrease in resources entering the marine food web.

This may be illustrated by a study from Fiordland in New Zealand, which consists of steep, forested fjords that experience heavy rainfall. LW and other plant matter frequently enters the fjords and sinks to the bottom. It was found that approximately 70% of hagfish (*Eptatretus cirrhatus*, a predatory fish) diet was derived from terrestrial vascular plants through various steps in the fjord food web *via* primary consumers of LW and other terrestrial vegetation (McLeod & Wing, 2007). Thus, a decrease or cessation of wood‐falls within brackish and marine environments has a direct effect on the woody nutrients available to detrital, benthic, and pelagic ecosystems (Hendy, Michie & Taylor, 2014; Nishimoto *et al*., 2021).

Like in rivers, sunken wood in estuaries can also create deposition zones where fine sediments settle on the sea floor due to the lower flow velocity. Some fish are attracted to these areas to conserve energy (Kim *et al*., 2016). In estuarine systems with ample rock available on the seafloor, the contribution of woody hard substrates is presumably less important than in entirely soft‐bottomed systems, where stable natural hard substrates are minimal (Dickson *et al*., 2023). However, as estuaries are the terminus point for sediment‐bearing rivers, most estuaries are generally soft‐bottomed (Emery & Stevenson, 1957; Göltenboth & Schoppe, 2006), and under natural conditions typically see frequent delivery of wood if woody vegetation is present within the watershed. Indeed, sunken wood tends to accumulate near river mouths (Schwabe *et al*., 2015).

In contrast to LW that has lost its branches and/or roots, root wads separated from the tree, colloquially known as stumps, cannot lie flat due to their inherent complexity. Consequently, much of the structure created by sunken root wads, and trees retaining root wads, remains elevated above the seafloor creating spatial heterogeneity and structural complexity. Furthermore, trees often grow roots around stones or even boulders which can remain in the root wad after the tree enters a river and be deposited in estuaries and at sea (Bennett, Doyle & Mather, 1996), increasing the variety of natural hard substrate around sunken wood. Darwin speculated that large rocks on coral islands had arrived due to transport while locked within driftwood (Darwin, 2009); this has been confirmed by critical observations (Bennett *et al*., 1996). These isolated stone reefs will remain on the seafloor after sinking and degradation of LW.

**Fig. 2:**
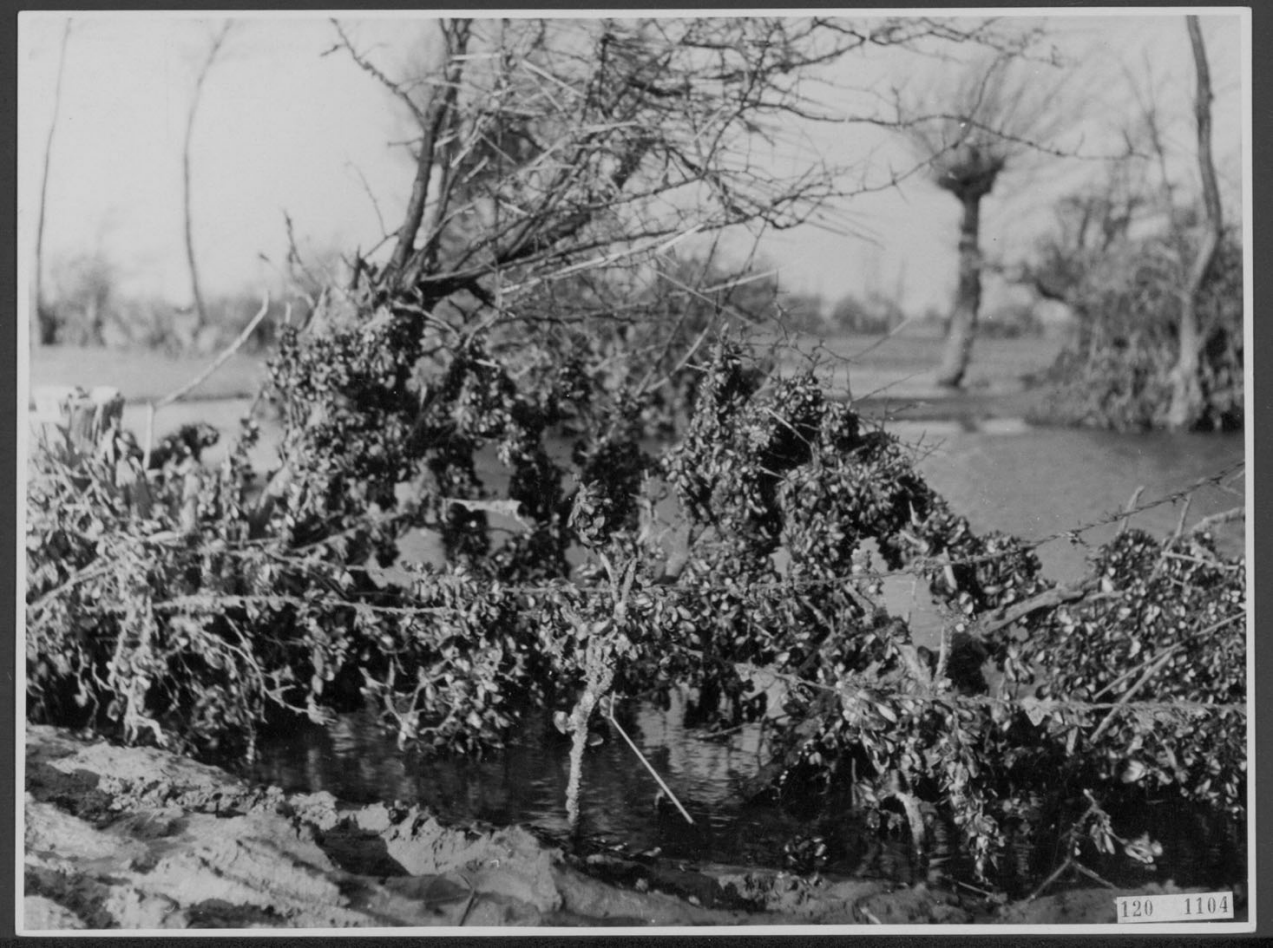
Mussels growing on a fruit tree, Zeeland, Netherlands, 1946 (Time Magazine, 1948).

Sunken wood provides important structural benefits in estuarine systems. Similar to its role in rivers, wood continues to act as an attachment substrate: not only for fish eggs and turf algae as in rivers, but also for macroalgae, barnacles, mussels, oysters and many other sessile plants and animals. This is notable in harbours where shellfish, such as oysters or mussels, grow on inter‐ and subtidal surfaces, including wooden pilings. The role of wood in estuaries as a settlement substrate for shellfish is perhaps best illustrated by a vivid example from World War II. The (now former) island of Walcheren, the Netherlands, located at the mouth of the Scheldt estuary, had its dykes breached by bombs in 1944 (DeGroot, 1997). When they were repaired over a year after the bombing, ‘*there had been mussels instead of fruit growing in the dead branches*’ of the fruit trees of Walcheren (Time Magazine, 1948, p. 257); see Fig. 2.

**Fig. 3:**
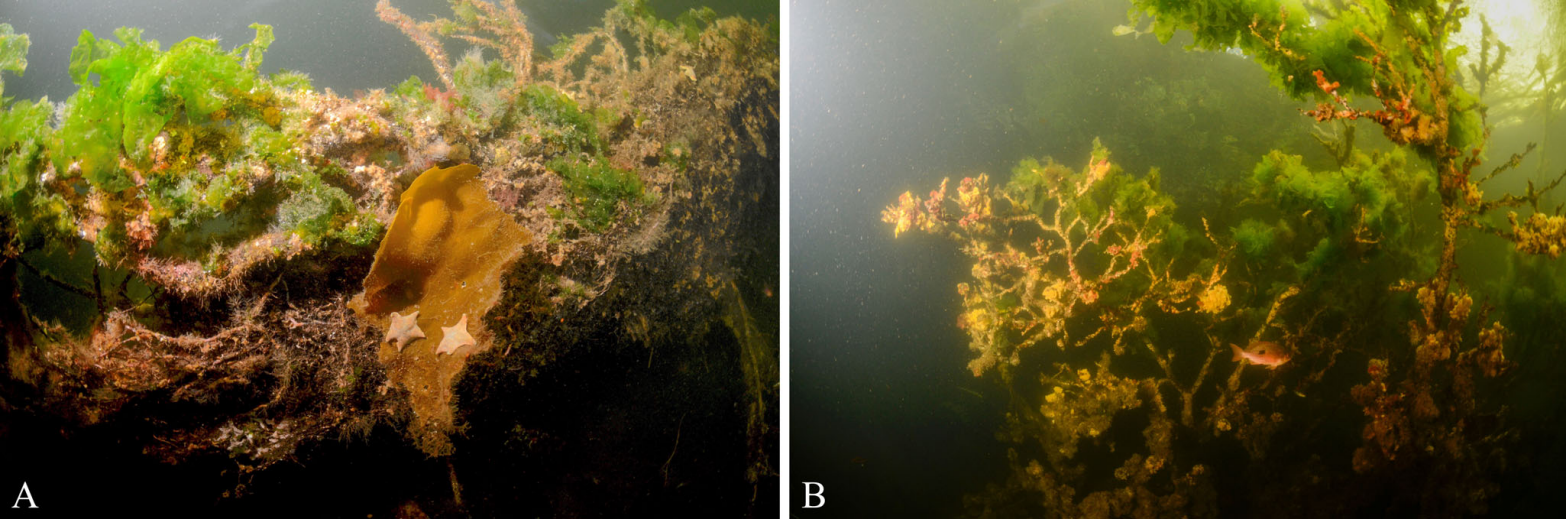
Sunken wood and associated communities, Fiordland, New Zealand. Photographs by Louise Bennet‐Jones.

Along with creation of hard substrate habitat, there are many additional ecosystem benefits of shellfish reefs, such as current and wave attenuation, water filtration, and the reduction of turbidity and eutrophication (Potet *et al*., 2021). In addition, LW containing marine wood borers, particularly shipworms, harbours higher species diversity, e.g. by providing nurseries for fish and octopod species in vacant shipworm tunnels of perished shipworms (Hendy *et al*., 2013, 2014). Furthermore, ecosystem benefits occur from attached macroalgae (and other organisms) that create zones of reduced wave and current energy, and provide additional habitat for egg attachment, foraging, shelter, and nursery functions for a variety of fish and invertebrate species (Fig. 3), as well as serving as a primary producer (Cotas *et al*., 2023; Dubi & Tørum, 1994).

As strange as it sounds, beavers play an underestimated role in natural estuaries and salt marshes throughout the North Atlantic and North Pacific oceans in North America (Hood, 2012) and likely was the case historically in Eurasia as well. Within the Skagit delta (Washington, USA), beaver dams and lodges have been found at equal or higher densities compared to non‐tidal rivers. These beavers quadrupled pool habitat for fish compared to undammed channels (Hood, 2012). Consequently, fish populations were found to be approximately three times greater in beaver‐dammed channels, as these channels did not fully empty at low tide as opposed to non‐dammed channels (Hood, 2012), allowing fish to shelter in these beaver channels at low tide. Thus, beavers and their propensity to use wood to dam channels enhances water retention, habitat complexity, and fish habitat within salt marshes and estuaries, as well as recruiting wood of all sizes to estuarine systems. This is (or should be) a strictly temperate Northern‐Hemisphere phenomenon; there are no Southern Hemisphere beaver‐equivalents, although beavers have been introduced in southern Argentina and have spread to southern Chile (Lizarralde, Escobar & Deferrari, 2004).

**Fig. 4:**
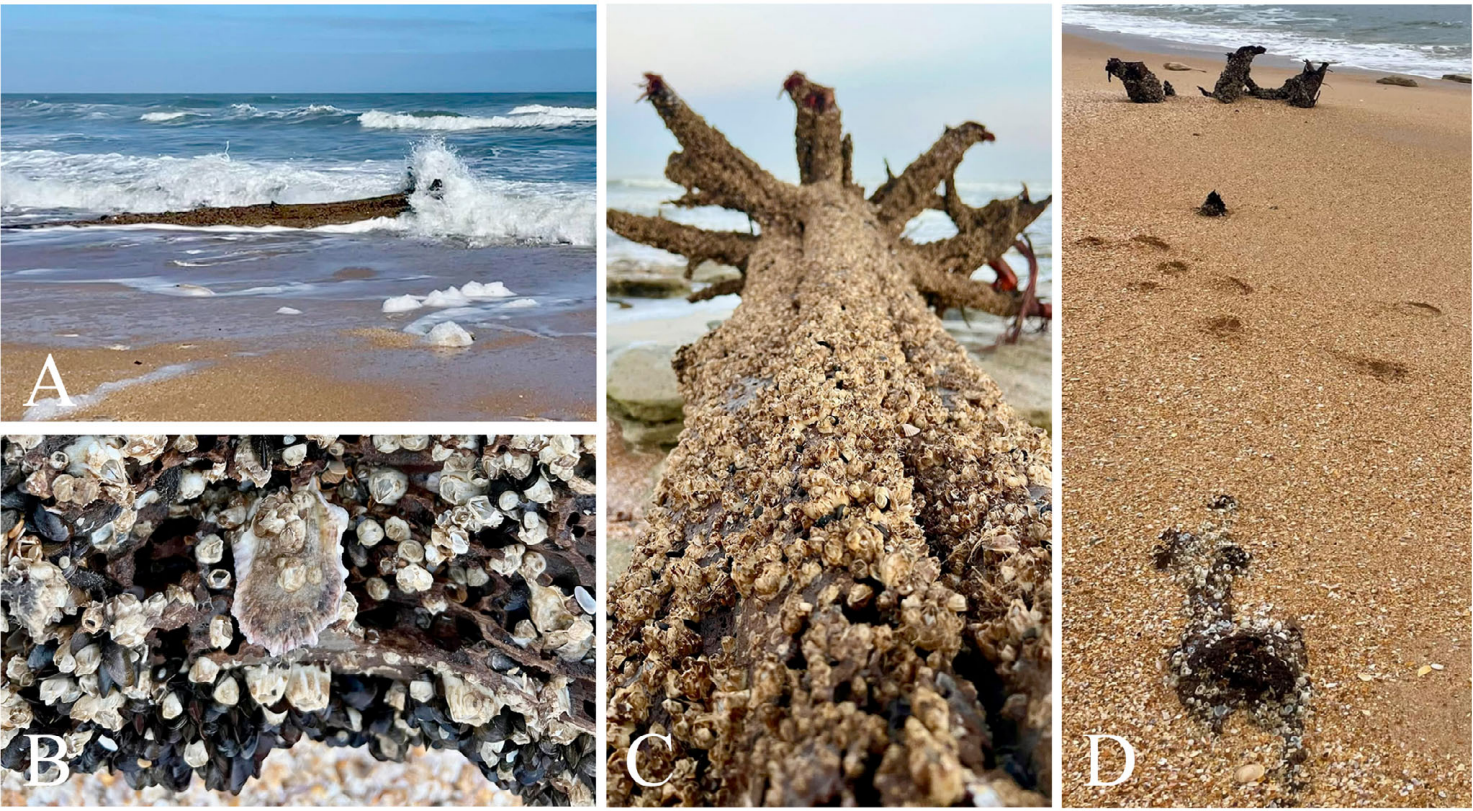
(A) Drift tree washes ashore. (B) When afloat, it was colonised by barnacles (top), oyster (middle), and mussels (bottom), and shows significant shipworm and gribble attack. (C) Driftwood beached at high tide. (D) Driftwood initiates dune. Photographs taken over 11 days in Florida, USA, by Terri Miller.

When buoyant, wood provides similar benefits to fish as when floating in rivers. As it stays afloat, it can be repeatedly deposited on shore by waves and high tides (Fig. 4A), leading to further abrasion of the wood by battering (Murphy *et al*., 2021). Driftwood beached beyond the high‐tide line can greatly influence beach morphology and beach profiles (Murphy *et al*., 2021), and initiate dune formation (Fig. 4D; Kramer & Wohl, 2015). It is also a trophic sea‐to‐land transfer, as deposited colonised driftwood allows terrestrial consumers to feed on marine biomass (Fig. 4B,C).

### (3) Estuaries – human impact

As in rivers, humans view driftwood as a generally negative feature in estuaries and coastal zones, although attitudes are changing (Hood, 2023). Some of this long‐standing attitude toward driftwood, snags, and deadheads manifests from the legitimate potential danger posed by LW to small boats (Doong *et al*., 2011, Terich, 2005). There is also a social aspect driving negative public attitudes; LW on beaches is generally seen as ‘ugly’ and ‘litter’ (Gall & Thompson, 2015). It is removed from beaches in many areas worldwide, particularly Europe. By eliminating beached driftwood, its ecological functions are terminated at an early stage. Given the decrease in wood export to sea (Wohl & Iskin, 2021), and its active removal across the entire riverine‐to‐marine gradient, it is clear that the land‐to‐sea wood linkage has dramatically decreased.

Not all human interaction with wood in estuaries involves removal; the opposite occurs on some rivers and estuaries large enough to allow mass transportation of wood. Here, huge additions of logged wood may occur in the form of log booms (Picard, Bornhold & Harper, 2013). This practice has taken place in France, Finland, Sweden, Italy, Austria, Poland, Thailand, and Brazil, and still occurs in present day Nigeria, Canada (British Columbia), USA (Alaska), and Russia (Labrecque‐Foy & Montoro Girona, 2023). A single log boom can contain hundreds to tens of thousands of logs chained together and is stored tied to nearshore pilings before being moved to sawmills for processing. In larger rivers near mills, such as the Fraser River passing through Vancouver, Canada, there may be thousands of log booms stretching hundreds of kilometers containing millions of logs, covering 40–50% of the river shoreline (Kussin‐Bordo *et al*., 2024). Occasionally, some of these logs break their chains and are lost to beaches or the sea, although loss of industrial wood from log booms has been greatly reduced since the early 1900s. Estimates suggest that historically, 3–8% of logs were lost during aquatic transport (Sarkar, 1984), while more modern estimates suggest that 15–50% of logs were lost during aquatic transport in the early 1900s (Labrecque‐Foy & Montoro Girona, 2023). These losses likely resulted in an increase in the amount of lost‐log driftwood on beaches in the Pacific Northwest of North America to a level higher than occurred naturally without human forestry activities. However, observations from a field ecologist in the San Juan Islands of the Pacific Northwest suggest that in places where beached log salvage was not possible, 85–99% of beached LW was of natural origin (Dayton, 1971). Dayton relates that these isolated beaches retained driftwood deposits of 30 to 75 m wide at the high tide line. Along with the addition of wood *via* industrial activities, wooden shipwrecks and wooden infrastructure also add wood to estuarine and marine environments, potentially providing habitat for wood‐boring species (Borges, 2007; Paalvast & van der Velde, 2011).

Such natural riverine LW is often trapped and removed, preventing it from entering the estuary and/or sea. For example, the Fraser River in Canada has a debris trap that accumulates approximately 25,000 to 100,000 m^3^ of wood during spring freshet; 90–95% of this is LW that has been recruited by natural processes, i.e. bank undercutting (Thonon, 2006). This is a marked difference *versus* Europe; for example, 40% of wood trapped in reservoirs on the Rhine is of human origin (Moulin & Piegay, 2004). Similarly, it is all removed. This variance in percentage of human‐created LW in the Rhine drainage basin *versus* the Fraser is likely due to intensive anthropogenic land use within the Rhine basin, while the Fraser basin is significantly less developed.

As established, wood borers can live in estuarine waters and therefore begin to consume wood here, although the majority of deterioration of LW by these organisms typically occurs in shelf seas. Nevertheless, they can render logs unusable for sawmills should the wood borers consumption of the logs continue for weeks to months. Therefore, log booms are moved back and forth between fresh and salt water by tugboat to kill shipworm. This can be problematic for nearshore ecological communities: log booms compact sediments and smother vegetation (Kussin‐Bordo *et al*., 2024) due to tidal action. Alternately, mill storage sites make use of freshwater influx in e.g., bays at the river mouth (Sedell *et al*., 1991). However, concentrating large amounts of wood in small bays may prove ecologically deleterious (Kussin‐Bordo *et al*., 2024; Washington State Department of Ecology, 2013). Bays which have been used historically for wood storage were found to hold log stacks piled nearly 10 m off the seafloor in places (Picard *et al*., 2013).

Mill sites in North America have long had the practice of disposing of wood chips, waste planks, bark, sawdust, and other miscellaneous wood waste into estuaries (Kirkpatrick, Shirley & O'Claire, 1998; Washington State Department of Ecology, 2013). Wood waste more than a few centimetres thick can smother established benthic and epibenthic organisms. Moreover, it compacts sediments and increases water turbidity, preventing seagrass colonisation and inhibiting algal growth (Washington State Department of Ecology, 2013). Large accumulations of waste wood are slow to decay and may persist for decades or longer (Kirkpatrick *et al*., 1998). When large whole logs are disposed of, sometimes a ‘bark tube’ may be left after the innards of the logs are consumed by marine borers (Kirkpatrick *et al*., 1998). These processes can lead to huge amounts of bark smothering the benthos. As waste wood products such as sawdust or woodchips provide a vastly higher surface area to volume ratio than an intact log, microbial decomposition can act on a large percentage of wood at once when compared to natural sunken logs. Consequently, decomposition of industrial wood waste can create high levels of chemical byproducts (sulfides, ammonia, and phenols) that can then enter the water column and/or sediments (Washington State Department of Ecology, 2013). Areas with good tidal flow show less impact from wood waste as opposed to areas with little to no tidal flow, where these byproducts can create hypoxic‐to‐anoxic zones that linger in the deeper water layers, leading to negative effects on aerobic organisms (Washington State Department of Ecology, 2013).

The coast of British Columbia, Canada, holds many sawmill sites; many of these locations disposed of or lost waste logs within the nearshore coastal waters. These bays, long used for wood‐handling, have significantly altered ecological communities when compared to natural nearby bays: indeed, the mill‐site bays, with numerous sunken logs, have many more sea cucumbers, lobsters, and anemones (Picard *et al*., 2013). These species require either attachment points to grow on or crevice complexity to evade predators (Picard *et al*., 2013); these requirements are fulfilled by sunken logs. Conversely, in natural nearby bays, crabs and sea stars were found to be five‐ and 25‐fold more abundant (Picard *et al*., 2013). Areas that receive unnaturally large amounts of industrial wood waste and/or wood storage may see very little to no establishment of macrobiotic communities (Washington State Department of Ecology, 2013). These case studies solely refer to temperate‐north sites; it is noted that specific literature on tropical and/or Arctic examples is lacking, and thus non‐temperate waters may see different ecological processes in such industrial wood‐handling sites.

## IV. SHELF SEAS AND COASTLINES

Shelf seas refer to an area of ocean from immediately adjacent to the coastline to the edge of the continental shelf, while their associated coastline extends from the low tide line to a variable distance inland, depending on the local geography and tides. For the purposes of this review, this can be considered the maximum distance that waves and associated wave‐mobilised buoyant objects – i.e. wave‐thrown LW – can reach on shore. Shelf seas can extend hundreds of kilometres offshore, although generally reach approximately 80 km offshore, averaging about 100 m deep (Simpson & Sharples, 2012). Shelf seas comprise less than 10% of the global marine surface area. They are influenced by terrigenous sediments from continental erosion of which they accumulate on average 15–40 cm every millennium (Simpson & Sharples, 2012). Parts of these coastal seas were once land that hosted extensive forests. As such, wood in coastal seas did not necessarily arrive from riverine transport; sunken forests are found in many places around the world such as the North Sea and Gulf of Mexico (Gontz, Maio & Rueda, 2013). Like terrestrial shifting sands in deserts, the seafloor does not necessarily stay static (Meijer *et al*., 2023): moving, burying and preserving these sunken forests, making them inaccessible to most organisms, only to expose them years to centuries later. Given that 70% of the Earth's seafloor is composed of soft sediments (Thrush & Dayton, 2002), geogenic and biogenic hard substrates are important islands of structure here.

### (1) Shelf seas and coastlines – transport, fate, and degradation

**Fig. 5:**
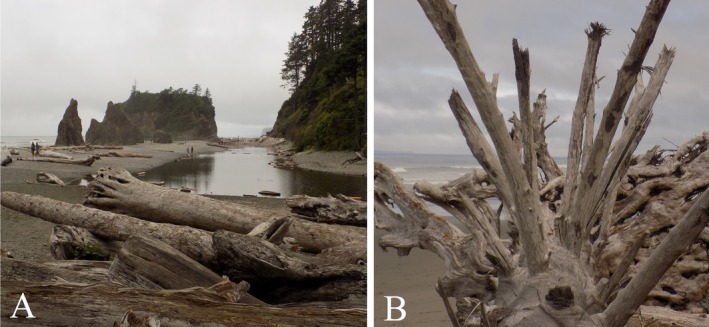
(A) Driftwood deposits on back beach. (B) Driftwood retaining significant structural complexity. Photographs from Washington, USA, by Ellen Wohl.

Like river systems where LW occurs in log jams and snags, coastal driftwood accumulates in heterogenous patterns – i.e. accumulation hotspots (Murphy *et al*., 2021). River mouths and pocket beaches seem to host the largest driftwood hotspots, but large deposits are also found on wide sandy back beaches (Fig. 5A) where it protects shorelines from waves (Kramer & Wohl, 2015). Along steep, rocky beaches, it is typically buried by sediment, pebbles, or cobbles, or re‐mobilised by the next storm (Terich, 2005). The latter can create large driftwood rafts (Doong *et al*., 2011; Murphy *et al*., 2021). These rafts can clog harbours, create navigational shipping hazards, smother coastal ecosystems, and damage infrastructure (Doong *et al*., 2011). When waves break on coastlines, floating logs can be launched dozens of metres into the air and thrown similar distances onto land; firsthand accounts report driftwood being thrown through living room windows during storms. Driftwood mobility is generally higher in winter when high energy and precipitation events are more frequent (Terich, 2005). Additionally, natural phenomena such as landslides and tsunamis can mobilise massive amounts of LW to shelf seas and open ocean (Doong *et al*., 2011; Carlton *et al*., 2017; Treneman *et al*., 2018; West *et al*., 2011).

Direct wind action (‘windage’) is a major cause of driftwood movement in coastal seas. However, the presence of root wads, bark, and branches have significant effects on wood transport at sea (Cornett *et al*., 2017). Like sailing vessels with sails deployed, low‐density driftwood, such as pine, that floats high in the water will present more surface area for the wind to act on. Conversely, a higher density wood, such as oak, will float lower in the water and sink more quickly (Ruiz‐Villanueva *et al*., 2016a). Some tropical woods such as ebony and rosewood have densities higher than that of sea water and therefore will not float at all (Randriamalala & Liu, 2010), or may shatter on impact upon falling (E. Wohl, personal communication, 2025). Additionally, buoyancy is influenced by several other factors, including the species of tree, the ratio of heartwood to sapwood, the presence of stones entangled in the roots, the degree of waterlogging and moisture content, root wads, branches, and the presence of biofouling organisms (Murphy *et al*., 2021). Furthermore, deterioration caused by crustacean wood borers (e.g. *Limnoria* spp., *Sphaeroma* spp., and *Chelura* spp.) on the wood surface and internal tunnelling by bivalve wood borers (e.g. *Teredo* spp., *Martesia* spp., *Lignopholas* spp.) can further alter buoyancy (Borges, 2007).

So long as wood remains buoyant, wave‐induced rip currents that extend well seaward of the surf zone provide a transit corridor for wood to escape offshore (Murphy *et al*., 2021). Using 1970s logging data from British Columbia, Canada, approximately 35% of wood that escapes the surf zone sinks within shelf sea areas, while another 25% returns to beaches or is exported to open sea; the remaining 40% was salvaged (Sedell *et al*., 1991). If we remove the salvaged floating logs from this total, more than half of lost timber sinks (Sarkar, 1984; Sedell *et al*., 1991), and this does not account for the LW exported to the open ocean. Hence, the cumulative figures of buoyant wood (that has escaped the surf zone) eventually sinking likely exceeds 50%. The rates of buoyant wood sinking are highly species dependent; thus, composition of wood afloat varies greatly by region (Thiel & Gutow, 2005b). It must be noted that the logs in the Sedell *et al*. (1991) study were industrial products undergoing shipment to mills and so had been stripped of branches, roots, and some bark, thereby generally decreasing overall density. The majority of the logs were western hemlock (Sedell *et al*., 1991), which is a relatively buoyant wood averaging 440 kg/m^3^ density (DeBell *et al*., 2004). Conversely, in the Amazon rainforest, the mean density of 268 tree species is 690 kg/m^3^ (Fearnside, 1997); Amazonian LW thus would sink more quickly than the British Columbia example. Furthermore, the density of ‘green’ (fresh or still living) and waterlogged wood is higher than average values reported in the literature (Ruiz‐Villanueva *et al*., 2016a). Indeed, pine blocks (420 kg/m^3^) used for a drift experiment were found to sink after 80 days (Carson *et al*., 2013). This indicates that estimated buoyancy lifespans of six to 17 months (Eggertsson, 1993) may be a significant overestimate for freshly downed trees.

When sunken, LW tends to accumulate in complex bathymetry such as canyons or gullies (Buhl‐Mortensen & Buhl‐Mortensen, 2018); individual chunks of sunken driftwood seem to occur, but literature on this is scarce. Anecdotal reports by amateur divers, bottom trawl fishermen and limited literature (Nishimoto *et al*., 2015) indicate that sunken wood in shelf seas exists, but the extent is unknown. Logically, there tends to be more near river mouths (Schwabe *et al*., 2015). Trawl records from the late 1800s show significant amounts of sunken wood and peat in the North Sea, loosely bordering present‐day coastlines (Olsen, 1883). This is likely a result of rising sea levels drowning forests rather than riverine deposition of LW. Regardless, the vast majority of global shelf seas have been bottom trawled (Amoroso *et al*., 2018; Kaiser *et al*., 2002) and hard substrates removed, indicating that acquiring an accurate historic baseline of sunken LW abundance and deposition may be impossible.

A study in shallow water off southern Spain attached 24 plates of six different materials to shipwrecks in waters of 7 and 12 m deep to assess the colonisation of biota. Of note, two of these materials were oak and pine; a hardwood and softwood, respectively. The oak panels showed less mechanical degradation and shipworm consumption compared to the pine panels (González‐Duarte *et al*., 2018). Interestingly, although the pine degraded more quickly, there was no significant difference in species found growing on the surface of the different panels.

Most coastal seas have higher salinity than estuaries (save for isolated near‐freshwater seas such as the Baltic); thus, wood borers are not constrained by salinity levels. The majority of the degradation and decomposition of LW in temperate shelf seas is carried out by shipworms and gribbles (Borges, 2007), and also xylophagaids at higher latitudes (Santhakumaran, 1984). In warm and tropical waters, pholads and pillbugs join shipworms and gribbles in the degradation of LW *via* biomechanical means (Borges, 2007). Chemoorganotrophic decomposition *via* fungi and bacteria is a concurrent but secondary process. That said, these microbes are important in ‘preconditioning’ the surface of LW for faster consumption by wood borers (Björdal, 2012). Should LW fall to the sea bottom and become buried, biomechanical degradation largely ceases, increasing the relative importance of microbial degradation.

Microbial activity will eventually shift the role of sunken wood away from being a hard, colonisable substrate for sessile species into a softer structure that will persist for years to decades – still suitable as habitat for biofilms, wood‐boring organisms and fish. Even after partial degradation and superficial microbial softening of the surface, LW continues to facilitate the accumulation of other hard substrate by creating deposition zones in the lee of the woodfall (Lehnert & Allen, 2002). Here, carcasses, bones, teeth, shells, gravels and any other small, mobile hard substrates may collect. This is borne out within the fossil record where remains of sharks, rays, skates, and bony fish, along with a number of invertebrates, including shipworms, are found in/around ancient sunken driftwood (De Schutter *et al*., 2023; Robin *et al*., 2018) in conditions similar to modern temperate sea bottoms.

**Fig. 6:**
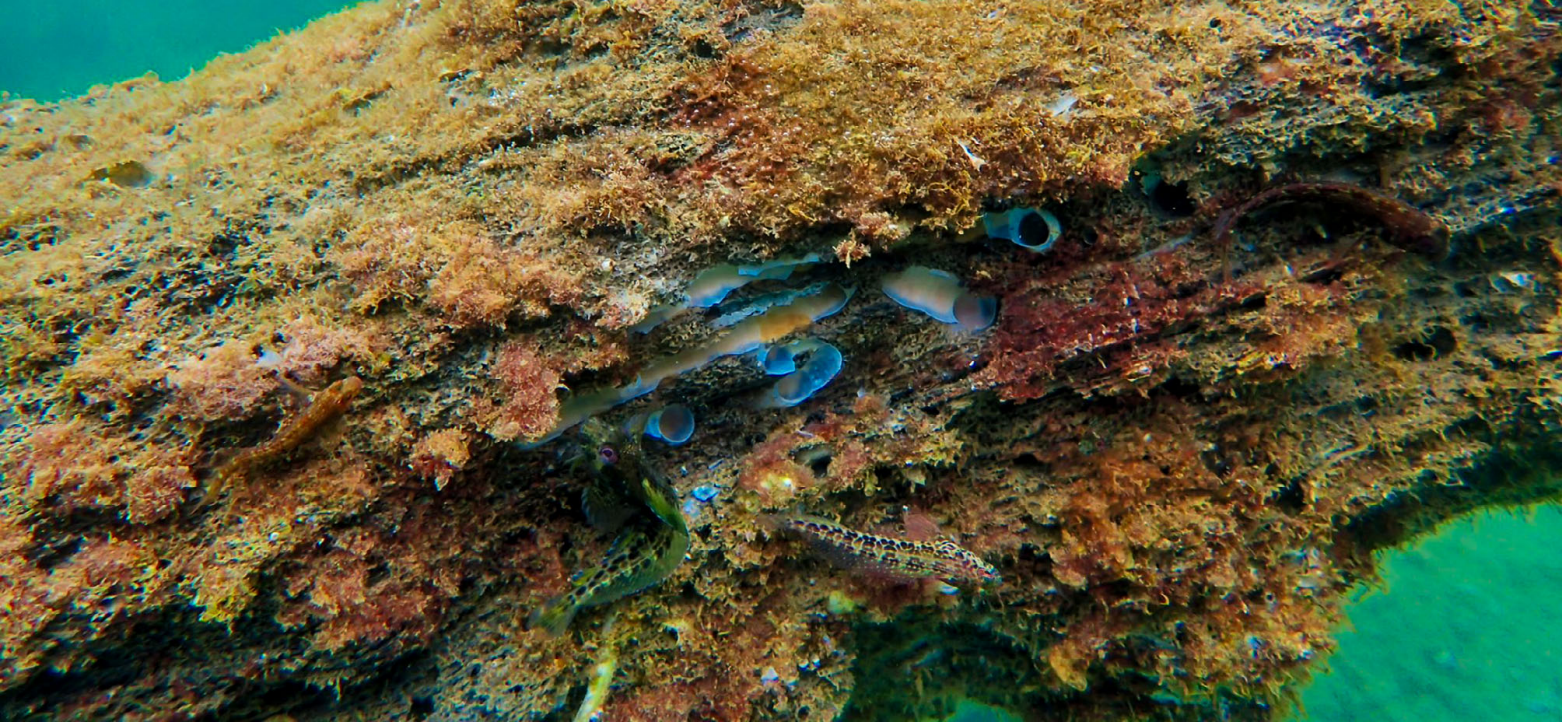
Sunken wood in Rio de Janeiro, Brazil showing exposed shipworm tunnels (centre) with calcareous lining; exterior of wood has been consumed by gribbles. Photograph by Laura Govers.

Experiments have shown that wood in the marine environment remains stable so long as the exterior remains inaccessible to wood borers, for example due to bark cover (Thiel & Gutow, 2005b), shellfish cover, or burial (Nishimoto *et al*., 2021). However, once this protective layer is removed, wood borers will start to bore and feed on wood. Crustacean borers (gribbles, pill bugs and chelurids) consume wood from the outside inwards. Conversely, bivalve borers (shipworms, pholads and xylophagaids) penetrate the wood and bore much deeper into the heart of woody debris and consume the innards while leaving the surface intact (see Fig. 6). Teredinids cover the internal walls of their tunnels with a calcareous lining to protect themselves from abrasion against the wood (Turner, 1966), but their tunnels never intersect. They continue to grow until the wood is exhausted and begins to break apart. In wood overcrowded with teredinids, it is common to observe stunted (dwarfed) individuals occupying the small remaining areas between tunnels (Turner, 1966; Borges *et al*., 2021). Crustacean borers typically feed more superficially, but as they consume surface wood, they can then access successively deeper regions of the wood, which has in many cases been internally weakened by shipworms, thus exposing their tunnels (Fig. 6). Once wood borers have largely consumed their shelter, their remains are rapidly consumed by predators and scavengers (Nishimoto *et al*., 2021), completing the land–sea nutrient linkage of wood.

Within mangroves, which are typically found in tropical and sub‐tropical regions, LW is subjected to microbial decay and wood‐borer degradation (Hendy *et al*., 2022; Kohlmeyer *et al*., 1995; Robertson & Daniel, 1989). While carbon export from mangroves is well documented for smaller organic fractions such as leaf litter and fine woody debris (Adame & Lovelock, 2011), the specific contribution of large woody material from mangroves to shelf sea and open sea habitats or marine carbon stores remains poorly quantified. Factors such as the buoyancy and high wood density of many mangrove species likely influence the potential for long‐distance transport and eventual deposition in other coastal zones (Rumbold & Snedaker, 1994). In contrast to the buoyancy period of most terrestrial‐source wood (6–17 months; Eggertsson, 1993), mangrove wood may remain buoyant for less than 1 day up to 1 week (Rumbold & Snedaker, 1994). Due to the short buoyancy time of mangrove wood, it is assumed that mangroves retain a large proportion of dead LW produced therein. However, sunken mangrove wood has been found ‘some kilometers’ offshore of mangroves by trawlers (Cragg & Aruga, 1987; Cragg, 1993), indicating at least some export to shelf seas, and potentially deep sea. Due to this presumed limited export in contrast to river and coastal delivered terrestrial‐derived LW, as well as inherently different biological processes within mangroves themselves, we only briefly touch on mangroves. We direct readers to the significant body of literature on the role of woody detritus and LW within these habitats (e.g. Krauss & Osland, 2019; Robertson & Daniel, 1989; Cragg, 1993; Romañach *et al*., 2018) for further reading.

### (2) Shelf seas and coastlines – ecological role

As in estuaries, buoyant LW continues to provide substrate, food, and shelter in coastal seas. Floating driftwood rafts in coastal and open seas rapidly develop their own sub‐surface ecosystem (Thiel & Gutow, 2005a). They serve as protection for decapod (e.g. shrimp and lobster) larvae against predating fish (Thiel & Gutow, 2005b) and create attachment substrate for eggs and sessile organisms (Figs 3, 4, 6) as well as cover for fish (Forget *et al*., 2020). Individual fish below floating rafts are often larger than conspecifics from open waters (Kingsford, 1993), while juvenile fish have a higher affinity for floating rafts than adults (Hunter & Mitchell, 1970). Overall, buoyant driftwood provides crucial habitat for not only coastal but also oceanic organisms, including microbial communities, invertebrates such as the oceanic teredinids *Teredora princesae* and *Teredothyra excavata* (Edmondson, 1963), along with ocean fish (Forget *et al*., 2020; Thiel & Gutow, 2005b), and macroalgae (Spencer *et al*., 2025).

Afloat, individual logs to large rafts of interlocked driftwood also provide a mode of transport for land‐based animals and plants (Gonor *et al*., 1988; Murphy *et al*., 2021). This facilitates movement and dispersal of organisms that are unable to swim or those that cannot swim long distances. Floating trees have been found to host organisms ranging from hydrozoans, sponges, barnacles, mussels, isopods and caprellids (Fig. 4B; Thiel & Gutow, 2005b; Treneman *et al*., 2018), to terrestrial vertebrates such as iguanas (Censky, Hodge & Dudley, 1998). More than 1200 species have been confirmed to use rafting as a dispersal method (Thiel & Gutow, 2005b; Carlton *et al*., 2017). When driftwood loses buoyancy, due to degradation, waterlogging and settlement, this may create the beginning of benthic sponge or shellfish reefs separated from existing hard substrates by long distances (Fig. 4B); this is demonstrated by chemoorganotrophic mussels living on hydrothermal vents that have used sunken wood as stepping stones to colonise hydrothermal vents scattered across both deeper waters and continental shelves (Distel *et al*., 2000; Bienhold *et al*., 2013).

Like sunken natural LW rafts, shipwrecks are known biodiversity hotspots (Consoli *et al*., 2015). A historic reconstruction of marine communities on a wooden 800‐year‐old shipwreck in Chinese waters revealed a fascinating pattern of micro‐biogeography and ecological succession within the sediments covering the ship (Li *et al*., 2021). These sediments were mainly of fine sand and organic mud. Correspondingly, soft‐bottom‐associated shellfish made up most of the 257 shellfish species found. Hard substrate‐associated shellfish were prominent within the sediment layers deposited shortly after sinking, but gradually declined within the sediment layers corresponding to time passing as the sediment deepened around the wreck. These findings indicate that the shipwreck's ‘artificial reef’ effect slowly weakened over time as gradually less of the wreck remained available for colonisation (Li *et al*., 2021). This demonstrates that woody hard substrates are used as attachment substrates (Nishimoto, Mito & Shirayama, 2009) until none is accessible due to burial or degradation. It is not hard to imagine woodfalls demonstrating a similar successional pattern as wooden shipwrecks. Indeed, fossils of shellfish living on sunken wood have been found dating back 200 million years (Kaim, 2011).

A Japanese study found that unique heterotrophic food webs can even form on small sunken wood chunks (Nishimoto *et al*., 2009); molluscs, annelids, spinculidians, cnidarians, nemertea, and arthropods were found in or on small sunken wood no larger than 41 × 11 cm at depths of 150–250 m. These chunks of wood also hosted wood‐boring organisms. As predatory worms or fish consume wood borers, energy that was locked in the wood enters the food web and is transferred to higher trophic levels (Nishimoto *et al*., 2021; McLeod & Wing, 2007). This example highlights that coastal sunken wood is both a direct and indirect source of food for a variety of animals as well as an attachment substrate for shellfish and other sessile species.

### (3) Shelf seas and coastlines – human impact

Although wooden shipwrecks form unique biodiversity hotspots, and areas such as the Pacific Northwest have seen notable marine timber input, direct human interactions with LW in the coastal zone are generally extractive and disruptive, e.g. by trawling, dredging, and coastal development/infill. Indeed, the vast majority (75%) of continental shelves (excluding Antarctica) have been bottom trawled: an area of approximately 20 million km^2^ (Kaiser *et al*., 2002). The degree of seabed disturbance depends on the type of trawl used. However, even lighter trawls can detach biogenic seafloor structures such as sponge reefs, deep water coral, or parts of shellfish beds (Thrush & Dayton, 2002) and, pertinently, LW reefs. Dutch trawlers in the North Sea routinely bring colonised LW back to port for disposal (J. Dickson, personal observation, 2024). Trawl efforts are often concentrated in the same area and can cover up to 100–700% of the same area annually (Watling & Norse, 1998). If the communities depend on hard substrate that has been removed, such as LW or glacial erratics (boulders that have been moved from their origin and deposited by glaciers), they cannot recover.

Once functionally important components of ecosystems have been disturbed, destroyed, or are missing, it becomes difficult to understand their ecological roles (Thrush & Dayton, 2002). Given that most shelf seas have been bottom‐trawled, often many times over, it is implausible to truly ascertain how trawling efforts have affected sunken wood and other hard substrates in shelf seas, and how this has affected the ecological roles of LW in coastal seas. However, we know that trawling is deleterious for endobenthic and hard substrate organisms, species which require stability: hard substrates provide some of that stability. Using shipwrecks as a proxy, species are found to be more abundant and diverse on and near wrecks compared with nearby sandy bottoms (Balazy, Copeland & Sokołowski, 2019; Consoli *et al*., 2015). Trawlers tend to avoid shipwrecks if possible to avoid gear entanglement, creating ‘*de‐facto* marine protected areas’ (Hickman *et al*., 2024). Large sunken logs may fulfill a similar role. As remaining LW in coastal seas is likely sporadic and patchily distributed, it can be assumed that at best, trawlers significantly disturb wood and its associated communities in coastal seas. At worst, wood and other substrates are removed along with its associated fish and reef communities; this, combined with the inherent seafloor disturbance, thereby eliminates important islands of structure, habitat and food in an otherwise generally homogenous ecosystem.

Acquiring a baseline of LW‐derived nutrient input to shelf seas is likely impossible given the extensive disruption of the global wood cycle. Yet, we know global forest‐to‐sea LW export flux has been reduced by 5 million m^3^ annually since pre‐landscape domestication (Wohl & Iskin, 2021), and this does not consider mangrove LW export. Globally, mangrove forests have declined by approximately 50% (Romañach *et al*., 2018) due to development, logging, and in particular, shrimp aquaculture (Hamilton, 2013). Inherently, it follows that mangrove‐derived LW export has also significantly declined. Thus, this loss of mangroves, and associated marine LW input, as well as severe decline in riverine and coastal export of LW (Wohl & Iskin, 2021) must have affected the conversion of LW by xylotrophic organisms into available nutrients for the marine food web. For instance, it was estimated that 1 m^3^ of softwood produces between 7 and 25 kg of shipworm biomass (Japan Fisheries Agency, 2006), but shipworms are only a fraction of all xylotrophic biomass present in the marine environment. It is very likely that the reduction of LW entering marine systems has strongly reduced terrestrial nutrient availability to primary marine consumers, in turn reducing the amount of prey available to secondary and tertiary consumers.

## V. OPEN OCEAN AND DEEP SEA

The open ocean consists of all marine waters past the edges of the continental shelves. With an average depth of approximately 3700 m (11,000 m at its deepest), the majority of the world's oceans exist in this open ocean region. The benthic regions of the open ocean are largely functionally ‘desert‐like’: flat, stable, and soft bottomed (Jørgensen & Boetius, 2007). However, isolated bathymetric features such as seamounts, hydrothermal vents, mud volcanos, cold seeps, canyons and trenches can induce up‐ and downwelling, nutrient cycling, and provide energetic resources for chemotrophic organisms (Jørgensen & Boetius, 2007). This in turn leads to higher biomass and biodiversity around these features (De Leo *et al*., 2010). Apart from these relatively spatially rare features in the deep ocean, hard substrate‐requiring animals on the abyssal plain largely rely on glacial drop stones and organic falls where whale, wood, fruit (such as coconuts) and other organic matter falls have reached the bottom (Bienhold *et al*., 2013; Pop Ristova *et al*., 2017). These organic falls provide both structure and lignocellulose nutrients to deep‐sea communities in a generally hard‐substrate‐depauperate environment (Bienhold *et al*., 2013; Wolff, 1979).

### (1) Open ocean and deep sea – transport, fate, and degradation

It is well established that rafts of various buoyant items, including driftwood, can reach the open ocean. Although observations of LW occur (Thiel & Gutow, 2005a), they appear geospatially haphazard – affected by wood density and degradation level, colonisation by marine wood‐boring species and other sessile organisms, proximity to large rivers, prevailing winds, and ocean currents such as gyres. Sunken wood is found in most deep‐sea trawls, suggesting that wood and other organics disperse far across ocean basins while positively buoyant (McClain & Barry, 2014; Turner, 1973; Voight, 2015; Wolff, 1979). This is supported by the presence of driftwood washing up on shores far from its source; for example, some driftwood on Hawaiian beaches has been identified to originate from trees in North America, the Philippines, Japan, and Malaysia (Gonor *et al*., 1988; Murphy *et al*., 2021).

Woodfalls in the deep sea occur in many locations but are especially prevalent in depressions such as trenches into which they are transported by turbidity flows (Anikouchine & Ling, 1967; Wolff, 1979). Wood was found in more than half of the bottom trawls conducted in the Caribbean and eastern USA at depths of 1800 to 5000 m (Wolff, 1979). Interestingly, wood found in the deep sea often still has bark and branches attached (Turner, 1973; Wolff, 1979); this is paradoxical given that rivers generally remove bark and branches from wood (Murphy *et al*., 2021). This indicates that some wood can reach the deep sea relatively undamaged, which can happen when wood enters rivers near estuaries, or directly into sea. This frequently occurs in mangroves (Hendy *et al*., 2022), as well as where forests and coastlines meet.

In contrast to estuaries and shelf seas, most teredinid species appear to be absent in the deep sea (deeper than 150–200 m). Instead, wood‐boring bivalves of the family Xylophagaidae (xylophagaids) are present generally from 200 to 7290 m (Turner, 1973). Nonetheless, some species of both families are known to have overlapping depth ranges, for example, specimens of *Nototeredo norvagica* (Teredinidae) were recovered at depths of up to 944 m (Turner, 1966), and *Xylophaga multichela* (Xylophagaidae) at 106 m (Voight, 2008). Additionally, in Norway, specimens of *X. dorsalis* have been found in the shallow subtidal (Santhakumaran, 1984). Xylophagaid wood borers opportunistically consume wood (Romano *et al*., 2014, 2020; Turner, 1973; Wolff, 1979, Voight, Cooper & Lee, 2020) with the assistance of endosymbiotic bacteria that live in their gills (Waterbury *et al*., 1983; Distel & Roberts, 1997). Despite the occurrence of other xylophagous taxa in woodfalls – e.g. *Munidopsis* lobsters and the echinoid *Asterechinus elegans* (McClain *et al*., 2025) – xylophagaids are the main mechanical degraders of sunken wood in the deep sea and make up most of the biomass of macrofaunal colonisers on LW here (Bienhold *et al*., 2013; Turner, 1973). They rapidly colonise sunken wood in large numbers and reproduce quickly (Bienhold *et al*., 2013; Wolff, 1979). These borers are present in deep oceans globally, apart from around Antarctica, as the Antarctic circumpolar current has an isolating effect that leads to the absence of wood there (Voight, 2015). In contrast to shallow‐water wood borers, xylophagaids were shown to bore through bark to all regions of the log, although de‐barked sections of the wood were most vulnerable (Bienhold *et al*., 2013). Notably, this study used Douglas fir (*Pseudotsuga menziesii*), a softwood with relatively low density (530 kg/m^3^). A study on *Acacia* spp. logs, a hardwood (770 kg/m^3^), shows that while wood was degraded by xylophagaids over 5 years, it still retained structural integrity and was not easily crushable (McClain & Barry, 2014). This is a marked contrast to sunken pine panels (350–500 kg/m^3^) that were ‘easily crushable by hand’ after 10 months submersion in the deep sea (Turner, 1973), illustrating the wood‐species‐dependent nature of wood degradation in the deep sea. Indeed, teak (*Tectona grandis*), a hardwood species (670 kg/m^3^), on the *Titanic* appeared to be relatively untouched by boring bivalves even after 70 years (McClain & Barry, 2014).

### (2) Open ocean and deep sea – ecological role

Like in shelf seas, floating LW provides a substrate for colonisation by numerous sessile organisms, creating a temporary ecosystem and food web in the ‘desert’ of the open ocean (Thiel & Gutow, 2005b). Also here, LW provides shelter for fish that use these rafts as foraging and hunting grounds as well as spatial reference points for navigation or schooling (Forget *et al*., 2020; Freon & Dagorn, 2000). Furthermore, LW directly provides nutrients (in the form of lignocellulose) to marine wood borers, and indirectly by generating its own ecosystem. When considering biogenic buoyant rafts in the open sea, it is important to note that these rafts are temporary; wood will eventually either sink to the bottom, break apart into small pieces due to mechanical battering or biological degradation, or eventually wash up on beaches.

Sunken to the seafloor, woodfalls provide similar ecosystem services in the deep sea as shelf seas and estuaries, although it is important to note that these woodfalls in the deep sea occur in a dark environment where (living) photosynthetic organisms are absent, eliminating the main source of primary production of most other ecosystems. Woodfalls provide lignocellulose, structure, hiding spots, and stable attachment points for sessile organisms, larvae and eggs. Due to the extreme nutrient deficiency of the deep open ocean benthos, the role of woodfalls as a resource is of greater relative importance than in shallow shelf seas (Bienhold *et al*., 2013; Schwabe *et al*., 2015; Turner, 1973; Wolff, 1979). Xylophagaids directly derive lignocellulose nutrients from sunken wood while other organisms such as cocculinid gastropods (limpets) and chitons probably feed on chemoorganotrophic bacteria and fungi growing on the woodfall (Wolff, 1979).

Overall, about 50 macrobenthic species, the majority of which are sessile, have been found to use deep sea sunken wood as a substrate, the most common of which are mussels (*Mytilus* spp.) (Turner, 1973). Polychaetes seek shelter under the bark (when present), while isopods are found in hollows in the wood (Wolff, 1979; McClain *et al*., 2025). Additionally, decapods, sipunculids, copepods, and gastropods have also been found under bark in wood retrieved in deep sea trawls (Wolff, 1979). Hence, the presence or absence of bark on sunken wood has not only a direct effect on mechanical decomposition by marine borers (Bienhold *et al*., 2013), but also has implications for physical niches that can be used as habitat in these woodfalls.

Finally, bacterial communities growing on wood in deep, low‐flow environments produce significant amounts of sulfide. These sulfides attract not only chemoorganotrophic fungi and bacteria (Björdal, 2012), and chemolithotrophic microorganisms that consume sulfides (Kalenitchenko *et al*., 2018), but also macroorganisms such as the symbiont‐containing hydrothermal vent mussel *Idas washingtonia* (Distel *et al*., 2000). Given the slow water circulation on the deep seafloor, fine wood chips and faecal matter accumulate around woodfalls and do not disperse beyond 1 m away (Bienhold *et al*., 2013). The enhanced respiration levels associated with the benthic woodfall community, combined with poor water mixing, leads to hypoxic conditions and the creation of sulfidic zones around these habitats (Bienhold *et al*., 2013; Pop Ristova *et al*., 2017; McClain *et al*., 2025). It is believed that hydrothermal vent mussels use these wood‐based sulfidic hotspots as stepping stones to colonise hydrothermal vents (Distel *et al*., 2000; Bienhold *et al*., 2013; Schwabe *et al*., 2015), like the terrestrial island‐hopping dispersal theory.

Pop Ristova *et al*. (2017) proposed that deep‐sea wood falls undergo five overlapping successional stages. The first is the ‘specialist stage’, in which wood‐boring organisms – particularly xylophagaid bivalves – colonise and initiate wood degradation (McClain & Barry, 2014). This is followed by the ‘opportunist stage’, during which the biomass of wood borers supports opportunistic detritivores, predators, and bacterivores (Bernardino *et al*., 2010; McClain & Barry, 2014). Thirdly, the ‘sulfophilic stage’, occurs as intensified cellulose degradation produces sulfidic conditions that sustain chemosynthetic organisms (Bienhold *et al*., 2013; Yücel *et al*., 2013; Kalenitchenko *et al*., 2015, 2016). In the subsequent, or fourth ‘senescence stage’, the wood‐fall assemblage declines with the progressive disintegration and dispersal of fragments, the last stage of wood degradation. However, in some cases, a terminal ‘reef stage’ may persist, whereby remaining structural elements provide habitat for species reliant on hard substrates (McClain *et al*., 2025).

### (3) Open ocean and deep sea – human impact

Direct interactions of humans with wood in the open and deep sea are limited, generally to isolated scientific studies (e.g. Bienhold *et al*., 2013; McClain *et al*., 2023; Romano *et al*., 2014). However, this could change with the onset of deep‐sea mining. Today, humans have created an altered marine system where buoyant garbage and industrial fishing gear mimics the structural role of bio‐ and geogenic rafts. In a pre‐anthropogenically altered world, rafts on the open sea consisted of driftwood, large buoyant fruits from coastal (i.e. *Cocos* sp.) and mangrove forests (i.e. *Xylocarpus* sp.), pumice, tar balls, seaweed, leaves, twigs, bark, or any other naturally buoyant debris that escaped the continental margins (Thiel & Gutow, 2005b). In the Anthropocene, these natural rafts have been supplemented with plastic, rubber, shipping containers, styrofoam, buoys, docks, ships, ghost fishing gear, active fishing gear such as fish attraction devices, and myriad other buoyant marine items (Thiel & Gutow, 2005b). Paradoxically, despite the human alteration of the landscape and rivers greatly diminishing LW output (Wohl & Iskin, 2021), open ocean ‘rafts’ have been increasing in size and number since the advent of plastic (Eriksen *et al*., 2014). However, these human‐made ‘rafts’ degrade slowly, if at all, and retain buoyancy far longer than wood (Häggblom, 1982). Instead of slowly releasing nutrients into the open ocean and abyssal plains as LW does, plastics do not fully degrade, accumulating and concentrating toxins (Barnes *et al*., 2009), nor do they provide lignocellulose nutrients as wood rafts do. In the open ocean and deep sea, the main difference between human‐made and natural LW rafts is that the latter floats for shorter time‐periods, hosts natural attachment surfaces of various micro‐rugosity, and provides lignocellulose‐derived nutrients to ecosystems both while afloat and on the seafloor.

## VI. CONCEPTUAL SYNTHESIS

### (1) Landscape and seascape modification by large wood

**Fig. 7:**
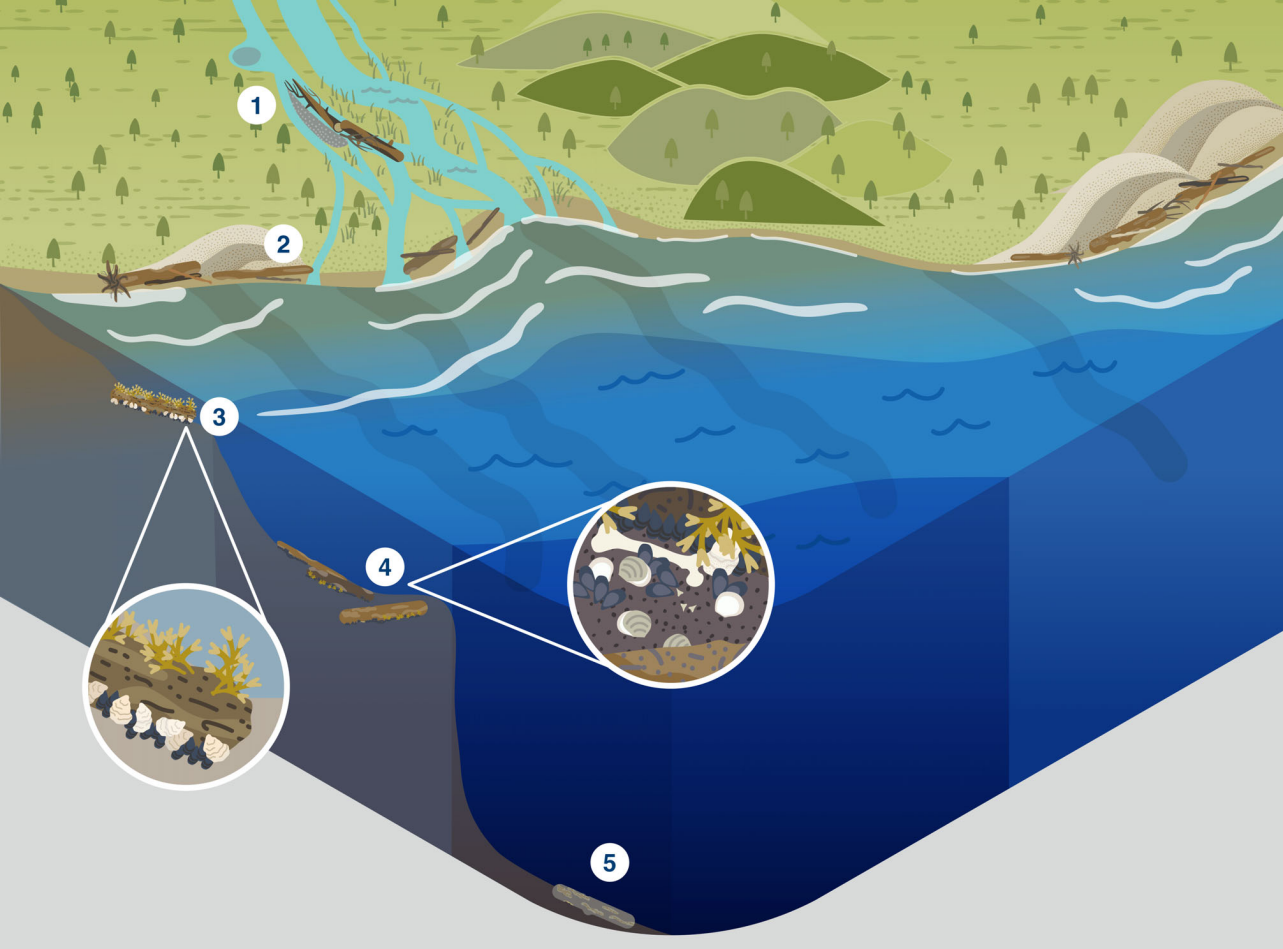
Landscape/seascape modification by large wood (LW). 1 = LW creates deep pools, meanders, side channels, and gravel deposition zones. 2 = LW protects shorelines against wave‐induced erosion. 3 = sunken LW and associated shellfish stabilise shifting sands. 4 = sunken LW accumulates other hard substrates. 5 = deep‐sea communities on LW change local biogeochemical seascape.

The ability of LW to modify its surroundings ranges from significant landscape‐level effects within river systems to highly localised geochemical effects in the deep sea (Bienhold *et al*., 2013); the smaller the geographic extent of the system, the more relative impact LW has. In riverine systems, LW creates log jams and is incorporated into beaver dams, both of which can create wetlands, new side channels, velocity shelters and sediment deposition zones, as well as provide shade, trap sediment, and slow river flow (1 in Fig. 7; Benke & Wallace, 2003; Lloyd *et al*., 1991; Ruiz‐Villanueva *et al*., 2016b; Wohl *et al*., 2019). Log jams in rivers create a potential positive feedback loop in which wood collects more wood, enhancing localised geomorphological effects until the wood jams rot out and disperse down river or are cleared by high‐water flow. Once within estuarine systems and along shorelines, LW rarely jams within the water but instead can sink or be deposited on shore. Here, it provides shoreline protection from erosion by waves, initiates dunes, and provides nutrients and shelter for plants and animals (2 in Fig. 7; Kramer & Wohl, 2015; Walker & Barrie, 2004). Subtidally, little is known about the geomorphological effects of sunken wood from estuaries to deep sea. Considering shipwrecks as a proxy, localised scour and deposition occurs in relation to prevailing currents (Balazy *et al*., 2019). Within estuaries, shelf seas, and the deep sea, sunken driftwood creates heterogenous habitat, providing patches of hard structure within largely soft‐bottom systems (3–5 in Fig. 7; Maser *et al*., 1988). Along with providing structure for organisms, sunken wood also brings woody nutrients to the deep sea, strongly influencing the local biogeochemical seascape around woodfalls (Bienhold *et al*., 2013; Pop Ristova *et al*., 2017).

### (2) Wood degradation

**Fig. 8:**
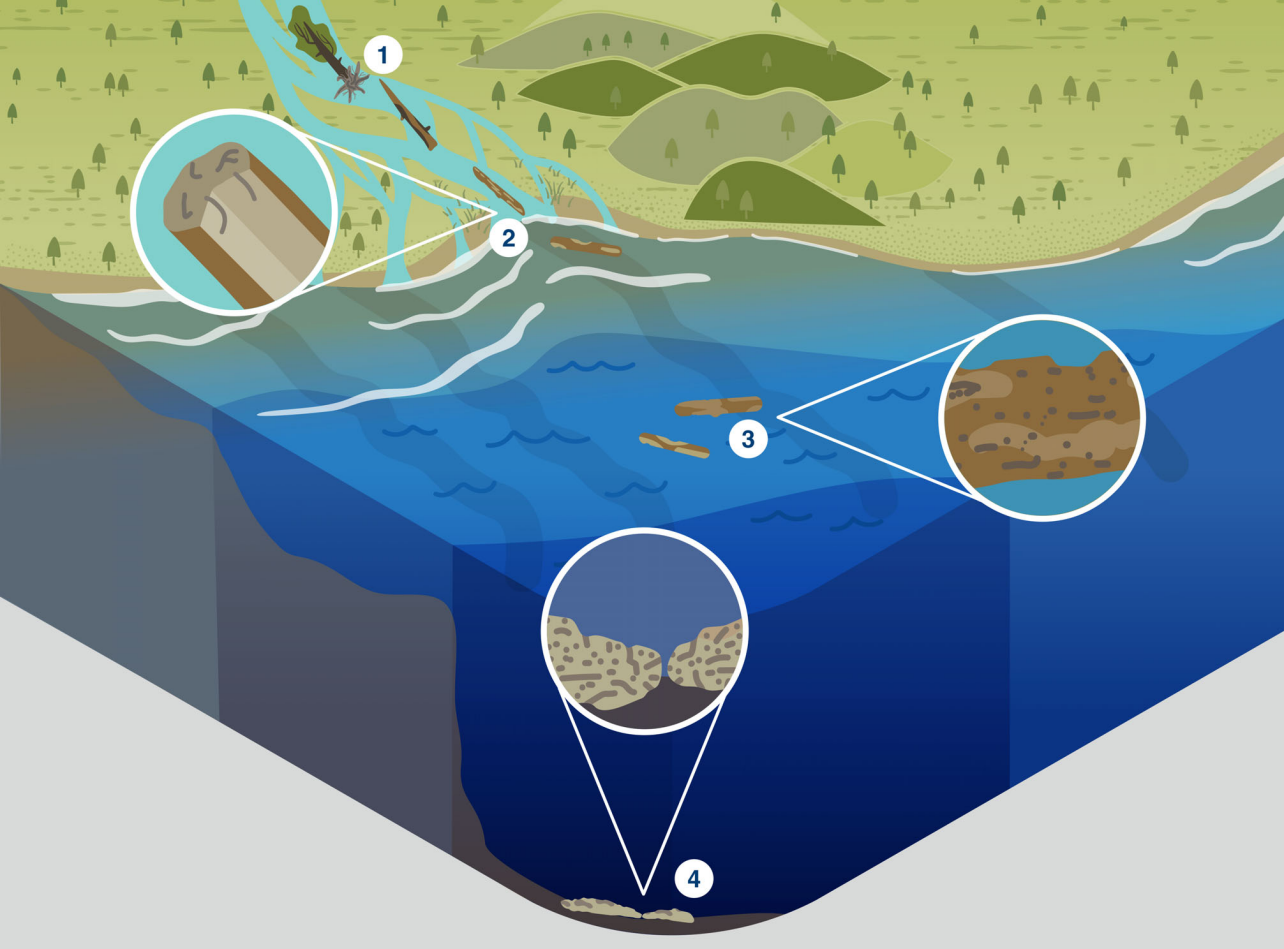
Processes of large wood degradation during transport from riverine to marine environments. 1 = leaves, bark, and smaller branches generally break off during flow down river. 2 = brackish waters allow presence of marine borers which enter the innards of the tree and create tunnels. 3 = afloat, marine borers continue to degrade wood. 4 = marine borers attack from all directions and (relatively) rapidly decompose wood into small pieces, with decomposition completed by microbes.

This review highlights a gradual transition from mechanical to biomechanical and chemotrophic wood degradation (Fig. 8). In rivers, mechanical degradation by abrasion and impacts is the primary degradation process. There is a loose correlation between the distance and time travelled by riverine wood and loss of finer structures such as bark, smaller branches, and finer roots (Murphy *et al*., 2021). Once reaching estuarine environments, rivers decrease in gradient, widen, and thus decrease in flow velocity and lose energy, while salinity increases permit some marine wood borers to begin biomechanical degradation. These organisms use their shells as a drilling device to create tunnels in the wood that shelter them both from the surrounding environment and predators. Therein, they can continue to grow, feeding on both wood and plankton that they filter using inhalant siphons through the opening created by the larvae on the wood surface (Fig. 8). Where wood has been stripped of its bark, marine microbes colonise and ‘soften’ the wood. Upon entering shelf seas, floating wood continues to be mechanically degraded when washed up against shorelines, but the main cause of degradation remains biomechanical by marine boring organisms, both afloat and sunken (Fig. 8; Cragg *et al*., 2020). However, the species of tree and/or presence of intact bark greatly determines the ability of marine borers to degrade wood (Borges *et al*., 2008; McClain *et al*., 2023). On the deep seafloor, where circulation is relatively negligible compared to near‐surface waters, woodchips and detritus produced by boring organisms remain relatively localised (Bienhold *et al*., 2013). Here, chemoorgano‐ and chemolithotrophic organisms finalise the degradation of wood once wood pieces are too small to be consumed by boring organisms.

Interestingly, degradation in freshwater systems always works from the exterior towards the interior of the wood. This outside–inward degradation generally ‘shrinks’ the log as successive layers are mechanically or biomechanically removed from the surface, while the core of the wood remains intact. This opposes the direction of degradation of wood in marine systems, which generally proceeds from the inside‐out, and then outside‐in due to wood borer‐specific activity. This process facilitates colonisation of organisms inside the wood, and simultaneously speeds up degradation, which amplifies the role of LW as a trophic resource while limiting its timespan as a settlement substrate.

### (3) Community effects

**Fig. 9:**
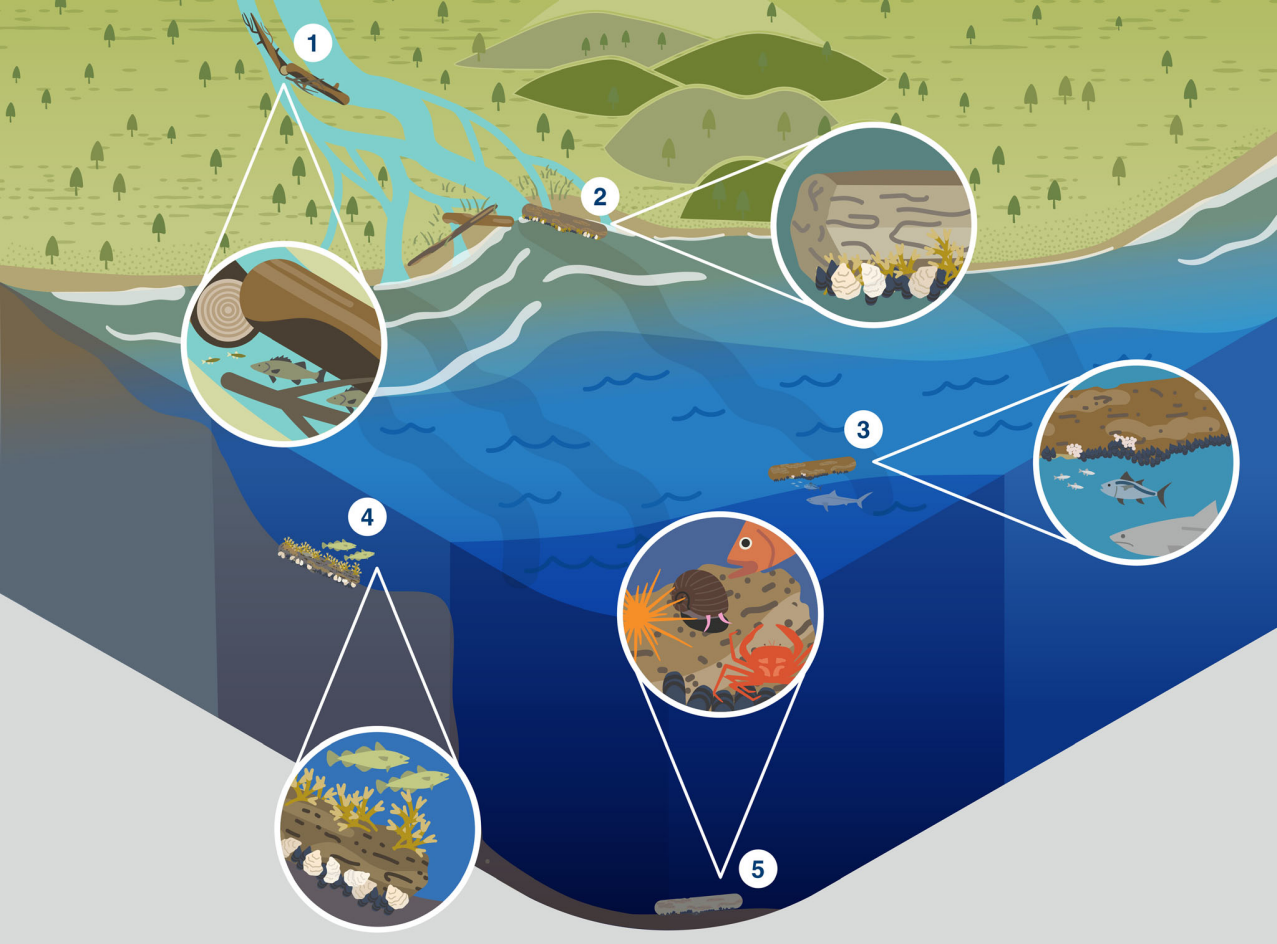
Effects of large wood (LW) on freshwater and marine communities. 1 = wood jams create low‐flow zones that are preferred fish habitat. 2 = brackish water allows shellfish colonisation of substrate; shipworm and gribble begin to colonise wood. 3 = LW floating rafts provide shelter and forage opportunities for fish, and a colonisation substrate for shellfish and other sessile organisms. 4 = sunken logs create attachment substrate for shellfish and seaweeds, and shelter and forage opportunities for fish. 5 = deep sea sunken logs provide energy, substrate, shelter, and foraging opportunities.

Ecological communities on and around aquatic wood within river systems largely rely on the abiotic changes induced by wood. Log jams and dams serve multiple functions such as creating velocity shelters (lees), as well as areas of cooler water and deep pools out of reach from terrestrial predators (1 in Fig. 9; Hafs *et al*., 2014; Lester and Boulton, 2008). LW is also an attachment substrate for eggs and larvae. Within estuarine systems, the focus shifts primarily to the LW itself: sunken wood acts as islands of structure within a soft‐bottom system, providing hard substrate for shellfish and macroalgae as well as lignocellulose nutrients for wood‐boring animals (2 in Fig. 9), thereby making wood‐derived nutrients available to higher trophic levels. In shelf seas and open ocean, floating rafts of LW provide cover from predators, shade, and attachment substrate for eggs and larvae (3 in Fig. 9). Here, small omnivorous fish and larger predators congregate around floating objects for both shelter and food. Once sunken to the seafloor, LW provides a stable attachment substrate for sessile organisms, shelter for a variety of benthic species, and lignocellulose nutrients for marine wood borers (4 and 5 in Fig. 9). This concludes the development of driftwood from a ‘fish attractor’ to potential biogenic reef foundation within estuarine, shelf, open, and deep seas, where wood can remain for many years before being completely degraded. Thus, the effect of wood on riverine communities is largely determined by habitat changes induced by wood, whereas the physical structure and nutrients of wood itself represent the most important factor to community assemblages in estuarine and marine systems.

### (4) Human interaction

**Fig. 10:**
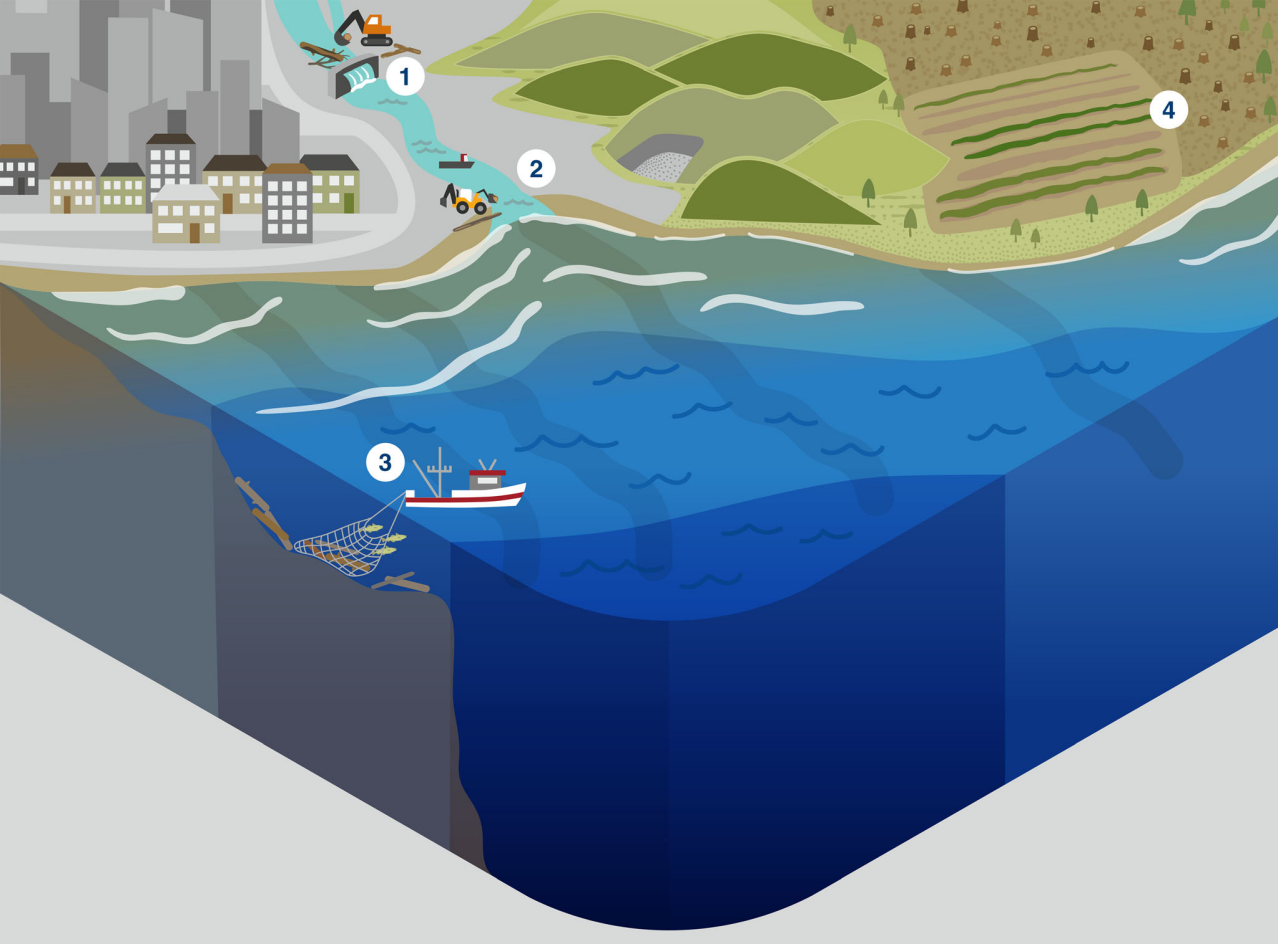
Impacts of human activities on natural large wood (LW) processes. 1 = dams and debris traps collect wood which is removed before sinking. 2 = wood in estuaries and on beaches removed. 3 = bottom trawlers remove sunken wood, hard substrate, and shellfish reefs. 4 = land use change from forest to agriculture eliminates sources of new LW.

Human activities have dramatically reduced wood within rivers and every environment seaward. Human–wood interactions in rivers are largely reflected by land‐use change (1 and 4 in Fig. 10) and river navigability. Where rivers are used for transportation, LW is removed from the system in developed regions. In estuaries and shelf seas, extractive activities such as dredging and trawling reduce LW availability (3 in Fig. 10). Reduced export from rivers has greatly decreased driftwood on coasts, with what little remains largely removed near populated areas (2 in Fig. 10). Apart from restricting its influx, human interactions with buoyant wood in pelagic shelf, open and benthic deep seas is limited. However, although humans have left the deep seafloor largely undisturbed thus far, this may change in the future with the advent of deep‐sea mining.

## VII. LARGE WOOD AND WOOD‐BASED AQUATIC RESTORATION

As shown herein, LW has an important role as an ecosystem engineer by influencing geomorphology and ecology from rivers to deep sea. Restoring historic levels of LW to rivers and the sea is implausible given the global landscape changes that humans have induced. However, smaller scale introductions of LW may prove useful to enhance ecosystem functioning and a valuable, cost‐effective method to increase ecosystem resilience in many aquatic habitats. Currently, the lack of consideration of the ecological and geomorphological roles of LW on coasts and at sea as opposed to rivers is surprising. Fossil records of sessile marine animals on sunken wood date back two hundred million years (Kaim, 2011), and wood fragments deposited over the past 19 million years in the Bengal Fan (Lee *et al*., 2019) show that wood moved to sea in vast amounts globally for epochs; this has been greatly lessened or halted in the Anthropocene due to human activity.

Restoration using LW seems to be following the path of water. Extensive work and research has been carried out in freshwater systems on the biological and geomorphological impacts of riverine LW, which increases channel stability and creates preferred habitat for fish and invertebrates (Easson, 2002; Hafs *et al*., 2014; Lester & Boulton, 2008; Whiteway *et al*., 2010). The biological and geomorphic role of LW on beaches and floating nearshore is understood, although not widely applied to coastal management. In these near‐coast regions, LW is largely assessed for its hazard potential (Doong *et al*., 2011; Murphy *et al*., 2021), with very little consideration given to the ecological and geomorphic implications of naturally occurring LW (Murphy *et al*., 2021). Research on the ecological role of placed estuarine and nearshore sunken wood is in its infancy with isolated experiments in Japan (Alam *et al*., 2020; Masuda *et al*., 2010) and the Netherlands (Dickson *et al*., 2023). In deeper shelf seas, open sea, and the deep sea, no applied research on LW has been conducted to our knowledge, while fundamental research on the behaviour and effects of LW has been sporadic across methodology, space, and time.

Although the long‐term nature of aquatic wood degradation (years to centuries) calls for multi‐decadal studies, it may be difficult to acquire funding and commitment. To our knowledge, the longest ongoing nearshore wooden reef monitoring continued for 4 years (Alam *et al*., 2020). In this period, the wood itself had not undergone notable degradation. Studies on wood in the deep sea involve less‐frequent monitoring, and the longest experiment we could locate lasted 5 years (McClain & Barry, 2014). However, this is not a problem unique to sunken LW. The majority (68%) of artificial reef studies are undertaken for 2 years or less (Lima, Zalmon & Love, 2019), while very few studies consider multi‐decadal timespans. One of the few that does was conducted in New South Wales, Australia, where fish richness and diversity on concrete artificial reefs significantly increased from the initial two‐year monitoring period to the 12th year (Becker *et al*., 2022), showing that artificial reef communities mature over longer periods than most studies consider. The role of biodegradable wooden artificial reefs over longer time frames is uncertain, warranting further research.

Aquatic and marine systems are under many threats, the most immediate of which are climate change and overfishing. Most of these threats – and changes – are clearly noticeable since they occur in real time. However, it is much harder to gauge the historic role of something that has almost disappeared – like wood. It is well established that humans have reduced global forest cover and wood transport to sea *via* river management and landscape change for hundreds to thousands of years. Bottom trawlers ply most coastal seas, disturbing the bottom and removing hard substrates along with fish. There is little widespread recognition of the historical presence of wood at sea, yet the mere existence of marine wood borers show that numerous individual marine species evolved to utilise terrestrial woody nutrients from estuaries to deep sea around the globe. The reduction, and in some places, near‐cessation of wood delivery by rivers and active removal of coastal wood clearly stands at odds with the historical situation of massive amounts of wood flowing to sea (Wohl & Iskin, 2021). It is conceivable that humans have damaged the marine environment by depriving it of vast amounts of LW‐derived structure and woody nutrients without realising it, but we cannot know the extent of these environmental degradations due to a heavily altered baseline of forests, rivers, and seafloors.

## VIII. MANAGEMENT RECOMMENDATIONS

We strongly suggest that riverine and estuarine managers begin to incorporate LW *at a sufficient scale* to stabilise and diversify riverine and nearshore (delta, estuary, coastal dunes) environments, re‐create heterogenous bottom profiles as habitat and spawning grounds, and release woody nutrients. Where possible, LW should be incorporated into shorelines and left in place/remain free to move; natural driftwood should no longer be removed unless it becomes a *realistic* safety hazard. We suggest that public education about the benefits and historic presence of driftwood is critical for societal acceptance of large woody ‘debris’.

Coastal managers should understand and acknowledge that driftwood plays an important role in shoreline protection, stabilisation, nutrient provision, and biodiversity. Placing sufficient LW *at scale* may prove an ecologically sound, cost‐effective, and natural method to augment hard coastal defences and support threatened coastal ecosystems – and human infrastructure – against rising seas. It is also a more environmentally sensitive practice than using imported large rocks or concrete structures to stabilise shorelines. We also recommend that coastal managers consider periodically (i.e. once every few decades) securing sunken LW at suitable spatial densities within nearshore and shelf sea bottoms to attempt to mimic the semi‐stochastic delivery of sunken wood that has occurred in the past.

Deep sea considerations are currently minimal, although deep sea wood input has certainly decreased concurrently with the decline of river‐exported wood, and so supplying LW at scale to the open and deep sea could prove beneficial for local ecosystems. Stochastic deposits of large wood in the open sea may partially account for missing LW habitat.

Overall, LW is lacking in all systems when compared to pre‐Anthropocene periods. Hence, we strongly suggest that managers pivot away from solely using ‘hard’ engineered solutions such as concrete and metal, and embrace cost effective, ecologically sound, and more carbon neutral approaches provided by LW: building with nature, for nature. We thus argue that wood re‐introduction can contribute to restoration along the entire freshwater–marine gradient, and hope that this review stimulates future research and management innovations.

## IX. CONCLUSIONS

(1) Large wood (LW) has been exported by rivers to sea for hundreds of millions of years. In rivers, LW attenuates and alters hydrodynamics, retains sediment and changes morphology. Along coastlines, driftwood anchors dunes, armours shorelines against erosion, provides lignocellulose‐based nutrients, and retains moisture. Estuarine, shelf, and open/deep seas hold both floating rafts and islands of sunken wood.
(2) Wood degradation gradually transitions from mechanical to biomechanical and chemotrophic. In rivers, degradation is primarily mechanical. In estuaries, salinity increases, allowing marine wood borers to begin biomechanical degradation. Their activity is the main degradation cause across brackish to marine environments. On the seafloor, chemotrophic organisms finalise wood decomposition as pieces become too small for boring organisms.
(3) LW accumulations act as biodiversity hotspots across the entire freshwater‐to‐marine gradient. In rivers, log jams and dams act as velocity and predation shelters, and create deep, cool pools, while the wood itself acts as an attachment substrate for eggs and larvae. From estuaries seaward, sunken wood provides nutrients for wood‐boring animals, and attachment substrate for sessile organisms such as bivalves and macroalgae. In shelf seas and open ocean, floating LW rafts provide cover from predators, shade, and attachment substrate for eggs, larvae, and sessile organisms.
(4) Humans have greatly reduced global forest cover and developed most rivers, estuaries, and many coastlines. These changes have led to a strong decrease in LW entering rivers, reaching estuaries, and moving to sea. In developed regions, rivers and coastlines cannot passively return to natural wood loads in a human‐dominated world.
(5) Restoring historic levels of LW to rivers and the sea is implausible but smaller scale introductions can enhance ecosystem functioning across the fresh‐to‐marine gradient. We argue that managers should consider incorporating LW reintroductions as restoration measures at scale to re‐diversify the freshwater‐to‐marine gradient.

## CONFLICTS OF INTEREST

None of the authors have a conflict of interest to disclose.

## Data Availability

Data sharing not applicable to this article as no datasets were generated or analysed during the current study.
